# Supplementary material for: Psychosocial singing interventions for the mental health and well-being of family carers of patients with cancer: results from a longitudinal controlled study
Source: BMJ Open. 2019 Aug 10;9(8):e026995. doi: 10.1136/bmjopen-2018-026995 (PMC6701813; doi:10.1136/bmjopen-2018-026995)
Supplement: Author's manuscript [file bmjopen-2018-026995.draft_revisions.pdf]

BMJ Open is committed to open peer review. As part of this commitment we make the peer review history of every article we publish publicly available.

When an article is published we post the peer reviewers' comments and the authors' responses online. We also post the versions of the paper that were used during peer review. These are the versions that the peer review comments apply to.

The versions of the paper that follow are the versions that were submitted during the peer review process. They are not the versions of record or the final published versions. They should not be cited or distributed as the published version of this manuscript.

BMJ Open is an open access journal and the full, final, typeset and author-corrected version of record of the manuscript is available on our site with no access controls, subscription charges or pay-per-view fees (http://bmjopen.bmj.com).

If you have any questions on BMJ Open's open peer review process please email info.bmjopen@bmj.com

BMJ Open

Psychosocial singing interventions for the mental health and wellbeing of family carers of cancer patients: results from a longitudinal controlled study

Journal:	*BMJ Open*
Manuscript ID	bmjopen-2018-026995
Article Type:	Research
Date Submitted by the Author:	01-Oct-2018
Complete List of Authors:	Fancourt, Daisy; University College London Research Department of Epidemiology and Public Health, Department of Behavioural Science and Health<br>Warran, Katey; The University of Edinburgh, Edinburgh College of Art<br>Finn, Saoirse; Royal College of Music, Centre for Performance Science<br>Wiseman, Theresa; The Royal Marsden NHS Foundation Trust, ; University of Southampton, Faculty of Health Sciences
Keywords:	Anxiety disorders < PSYCHIATRY, MENTAL HEALTH, Depression & mood disorders < PSYCHIATRY, SOCIAL MEDICINE, ONCOLOGY

SCHOLARONE™ Manuscripts

For peer review only

Psychosocial singing interventions for the mental health and wellbeing of family carers of cancer patients: results from a longitudinal controlled study

SHORT RUNNING TITLE: Psychosocial singing interventions for the mental health of carers

Fancourt D[1]*, Warran K[2], Finn S[2], Wiseman T[3,4]

[1] Department of Behavioural Science and Health, University College London, UK

[2] Centre for Performance Science, Royal College of Music, London, UK

[3] The Royal Marsden NHS Foundation Trust, London, UK

[4] Faculty of Health Sciences, University of Southampton, Southampton, UK

* Corresponding author: Dr Daisy Fancourt, University College London, 1-19 Torrington Place, London WC1E 7HB, d.fancourt@ucl.ac.uk

Word count: 2099

For peer review only - http://bmjopen.bmj.com/site/about/guidelines.xhtml

ABSTRACT

Objective. The mental health challenges facing people who care for somebody with cancer are well documented. While many support interventions focus on the provision of information or cognitive behavioural therapy, the literature suggests that psychosocial interventions could also be of value, especially given the low social support frequently reported by carers. Singing is a psychosocial activity shown to improve social support, increase positive emotions and reduce fatigue and stress. This study explored the effects of weekly singing on anxiety, depression and wellbeing in carers over a six-month period.

Design. A multi-site longitudinal controlled study

Setting. The Royal Marsden Hospitals in Greater London.

Participants. 62 adults who currently care for a spouse, relative or close friend with cancer who had not recently started any medication or psychological therapy.

Interventions. On enrolment, participants selected to join a weekly community choir for 12 weeks (n=33) or continue with life as usual (n=29).

Outcome measures. Primary outcome was mental health using the Hospital Anxiety and Depression Scale. Secondary outcome was wellbeing using the Warwick Edinburgh Mental Wellbeing Scale. Using linear mixed effects models, we compared the change in mental health and wellbeing over time between the two groups while adjusting for confounding variables including demographics, health-related variables, musical engagement and length of time caring.

Results. Participants in the choir group showed a significantly greater decrease in anxiety over time than participants in the control group (B=-0.94, SE=0.38, p=.013), and a significantly greater increase in wellbeing (B=1.25, SE=0.49, p=.011). No changes were found for depression. Sensitivity analyses showed carers with anxiety or below-average wellbeing were most likely to benefit.

Conclusions. This study builds on previous research showing the mental health benefits of singing for people with cancer by showing that weekly singing can also support anxiety and wellbeing in carers.

KEYWORDS

Cancer, oncology, carers, anxiety, depression, wellbeing, singing, psychosocial, social support

For peer review only - http://bmjopen.bmj.com/site/about/guidelines.xhtml

ARTICLE SUMMARY

Strengths and limitations of this study

- We used linear mixed effects models to compare the change in mental health and wellbeing over time amongst carers who did and didn't take part in a weekly choir over 24 weeks.
- Our analyses adjusted for confounding variables including demographics, health-related variables, musical engagement and length of time caring.
- This study had many strengths including its use of validated measures of mental health and wellbeing, its longitudinal tracking of effects of multiple time-points and its controlled design.
- Our groups were well matched at baseline and results were not attenuated by the consideration of baseline confounders.
- However, our results were found in a relatively modest sample size, so future studies are required to ascertain whether results can be replicated in larger samples.

For peer review only

For peer review only - http://bmjopen.bmj.com/site/about/guidelines.xhtml

INTRODUCTION

Family members and close friends, particularly spouses, are often primary caregivers for individuals affected by cancer, playing a vital role in their care process. Yet, well documented across the literature are the psychological, physical, social and economic challenges faced by informal cancer carers [1,2]. In relation to *mental health*, carers have repeatedly been found to have greater anxiety and depression levels than general population controls [3,4]. Indeed, psychological distress in carers correlates with the distress experienced by patients and has been found through meta-analysis to be no different in size [5,6]. Prevalence rates of depression are around 26% in both patients and spouses and the prevalence of anxiety is around 28% in patients and 40% in spouses [7]. In particular, anxiety has been found to increase when caring for somebody with advanced cancer, exceeding depression in carers [8]. This has led to suggestions that anxiety, rather than depression, is the largest psychological problem in carers and calls for interventions that can tackle it [7].

In addition to *mental health* challenges, caring for somebody with cancer has also been found to affect *wellbeing*. A Europe-wide survey has found mental as well as physical health-related quality of life to be significantly lower in carers than non-caregivers [9]. Female carers in particular report lower quality of life than women who are not carers [5], and quality of life for carers of both genders has been found to vary across the illness trajectory [10]. Given that wellbeing is associated with mental health and also related factors such as resilience and coping, there is a need too for interventions that can support wellbeing in carers.

Many interventions for carers focus on the provision of information designed to directly support carers in their roles, or involve cognitive behavioural therapy to support enhance coping [11,12]. A meta-analysis of therapeutic counselling, skills training and psychoeducational interventions for caregivers of cancer patients found evidence of reductions in caregiving burden, enhanced coping strategies, self-efficacy, physical functioning, anxiety, family relations and social functioning [13]. Group delivery and caregivers attending alone were identified as moderators of these effects. However, there has also been increasing interest in the role of psychosocial interventions for supporting mental health and wellbeing in carers (e.g. Badr & Krebs, 2013). Such interest has been encouraged by research suggesting that, in addition to specific needs relating to their caregiving, carers frequently have unmet personal needs and low social support themselves [15]. In a population-based study of family carers of cancer survivors, social support was one of the strongest predictors of quality of life [16]. Carers with less social support also have a higher

For peer review only - http://bmjopen.bmj.com/site/about/guidelines.xhtml

risk of developing depression and anxiety [1]. Yet, caregivers often feel that they receive less support in comparison to how much patients feel that they receive [16].

Singing is a promising psychosocial intervention that has been used to support mental health and wellbeing in diverse populations including amongst cancer patients [17]. Singing is a multimodal intervention that has been shown in cancer caregivers to increase perceived social support, enhance short-term positive emotions, reduce fatigue, promote relaxation and also reduce levels of stress hormones and mood-related inflammation over a single singing session [18]. However, to date there is little research into whether singing can specifically reduce anxiety and depression and enhance wellbeing in cancer caregivers over longer periods of time. Consequently, this study explored the effects of weekly singing on anxiety, depression and wellbeing in carers of somebody affected by cancer over a six-month period.

MATERIALS AND METHODS

Participants and procedure

This study was a multi-site longitudinal controlled study. To be eligible, participants had to self-identify as currently caring for a spouse, relative or close friend with cancer. We excluded members of hospital staff in order to differentiate between formal and informal care. Participants were excluded if they were under the age of 18, if they were already engaged in a weekly group choir, if they had started a formal course of psychological therapy in the last month or were scheduled to start one in the next 12 weeks, if they had started any new medication for anxiety or depression in the last month, or if their level of English was insufficient to complete the questionnaires required. Participants were recruited through NHS hospital trusts in Greater London, carer support groups, community and charity events and social media. A total of 396 were screened for enrolment, 66 were eligible and consented to take part, and 62 participants (29 control and 33 experimental) enrolled in the study (see Figure 1 for CONSORT diagram of participants). The study received ethical approval from the National Research Ethics Service [16/LO/0579] and all participants provided informed consent.

FIGURE 1 ABOUT HERE

Figure 1: CONSORT diagram of participants

For peer review only

For peer review only - http://bmjopen.bmj.com/site/about/guidelines.xhtml

On enrolment, participants were given the option of joining a weekly choir for 12 weeks. Participants who did not select to join a choir formed the control group and continued with life as usual. Participants who did select to join a choir took part in a 90-minute session each week led by a professional choir leader, of which 60 minutes was dedicated to singing (including warm-up exercises, learning new songs and singing familiar songs) and 30 minutes to socialising. These choirs were led by Tenovus Cancer Care, specifically for people affected in some way by cancer, and the format of the sessions has been described in previous studies [19]. Following 12 weeks in the choir, participants were given the option to leave the choir, with 19 participants (58%) choosing to leave and the remaining 42% choosing to continue attending. All participants were asked to complete questionnaires at baseline, 6 and 12 weeks as well as at 3-month follow-up (24 weeks).

Patient and Public Involvement

The research questions and outcome measures were selected with the support of a PPI group who were participants in the Tenovus Cancer Choirs in Wales. These participants also approved the design of the study and the decision to run this as a longitudinal controlled rather than randomised study. Participants in the study also supported recruitment efforts by helping to identify further participants. Dissemination plans include presentations back to members of all of the Tenovus Cancer Choirs in Wales and England.

Measures

Our primary outcomes were anxiety and depression, measured using the Hospital Anxiety and Depression Scale (HADS), ranging from 0-21 for each construct with higher scores indicating poorer mental health (Bjelland, Dahl, Haug, & Neckelmann, 2002). Scores of 0-7 are considered as non-indicative of anxiety or depression, while scores of 8-10 present mild cases, 11-14 present moderate cases, and 15-21 present severe cases.

Our secondary outcome was wellbeing, measured using the Warwick Edinburgh Mental Wellbeing Scale (WEMWBS) short form, which encompasses both hedonic and eudemonic wellbeing [20]. The scale is scored from 7-35 with higher scores representing higher levels of wellbeing. As recommended in validations, the raw scores were logit transformed prior to analysis. The New Economics Foundation suggests five levels of wellbeing based on quintile analyses of data in the UK Understanding Society Survey, 2009: poor (<22), below average (22–24), average (25–26), good (27–28) and excellent (29– 35).

For peer review only

For peer review only - http://bmjopen.bmj.com/site/about/guidelines.xhtml

Additionally, we collected data on age, gender, income (<£16,000, £16,000-£30,000, £31,000-£60,000, £61,000-£90,000, >£90,000), employment status (unemployed, voluntary/temporary work, part-time work, full-time work, retired), whether participants were receiving psychological therapy, whether participants had any other health condition, whether participants had previously sung in a choir, how confident participants felt about singing (from 1-not at all to 5-very), whether participants had attended a music concert or performance in the past year, whether they had taken part in any other music activity in the past year, and how long participants had been in a caring role for.

Statistics

To compare the choir vs the control group at baseline, we used one-way analyses of variance and chi-square tests. To explore changes in mental health and wellbeing over time and between groups, we used linear mixed effects models (LMMs). LMMs allow for natural heterogeneity amongst participants and, unlike some other repeated measures analysis models such as repeated measures analyses of variance which deal with missing data through list-wise deletion, linear mixed effects models make full use of the dataset. Our participants provided data an average of 3.4 times across the four time points, providing 180 data points. Our models used a random intercept and random slope, with an unstructured covariance matrix of the random effects. We modelled time both as a continuous variable and as four separate time points. We tested quadratic models but found no evidence to support their usage over a linear model. All models demonstrated normality of residuals and no evidence of heteroscedasticity.

We built up our final model through considering nested models of relevant covariates. Model 1 was unadjusted, model 2 adjusted for demographic variables, model 3 additionally adjusted for health-related variables, model 4 additionally adjusted for cultural engagement and attitudes to singing, and model 5 additionally adjusted for length of time caring for somebody with cancer. We used the log-likelihood ratio, Akaike's information criterion and Bayesian information criterion to confirm best fit of model 5. We calculated margins of response from the fully-adjusted model and created profile plots to illustrate the time*group interactions. Sensitivity analyses explored whether those with poorer mental health and wellbeing at baseline experienced the same responses as those with higher levels of mental health and wellbeing at baseline, and also considered whether singing patterns after the initial 12 weeks affected outcomes at 24 weeks. All analyses were conducted using Stata v14.

RESULTS

Demographics

For peer review only

Participants were well matched at baseline on all demographic variables including mental health and wellbeing. The only significant difference between groups was in confidence in singing, with the control group showing lower overall confidence than the singing group (see Table 1).

Table 1: Baseline demographics of participants

	Control (n=29)	Choir (n=33)	P value
Age, mean (SD)	51 (15)	58 (14)	0.075 [a]
Sex, % female	72.4%	81.8%	0.38 [b]
Income, %			0.077 [b]
<£16,000	12.0%	20.7%	
£16,000-£30,000	24.0%	41.4%	
£31,000-£60,000	32.0%	24.1%	
£61,000-£90,000	32.0%	6.9%	
>£91,000	0%	6.9%	
Employment status, %			0.20 [b]
Unemployed	13.8%	3.2%	
Voluntary	0%	6.5%	
Part-time work	20.7%	29.0%	
Full-time work	37.9%	22.6%	
Retired	27.6%	38.7%	
Currently having therapy, %	10.3%	24.2%	0.15 [b]
Health condition, %	72.4%	60.6%	0.33 [b]
Previously sung in a choir, %	65.5%	48.5%	0.18 [b]
Confident in singing, %			**0.042** [b]
1 – not at all	50%	21.2%	
2	21.4%	12.1%	
3	21.4%	36.4%	
4	3.6%	21.2%	
5 - very	3.6%	9.1%	
Attended a concert or performance in the past month, %	32.1%	51.5%	0.13 [b]
Took part in a music activity in the past month, %	18.5%	36.4%	0.13 [b]
Length of time being a carer, %			0.38 [b]
0-3 months	0%	9.7%	
3-12 months	17.9%	19.4%	
1-3 years	50%	35.5%	
3-5 years	14.3%	22.6%	
5+ years	17.9%	12.9%	
Anxiety, mean (SD)	8.04 (4.71)	8.97 (4.19)	0.42 [a]
Depression, mean (SD)	5.43 (4.49)	4.88 (3.08)	0.57 [a]
Wellbeing, mean (SD)	23.15 (4.36)	21.71 (3.70)	0.17 [a]

[a] one-way ANOVA; [b] chi-square test

Anxiety

At baseline, 44.3% (n=27) of participants showed no anxiety (HADSA score 0-7), 21.3% (n=13) showed mild anxiety (HADSA score 8-10), 23.0% (n=14) showed moderate anxiety (HADSA score 11-14), and 11.5% (n=7) showed severe anxiety (HADSA score 15-21). The linear mixed effects model showed that there was a significant time by group interaction, with participants in the choir group showing a significantly greater decrease in anxiety than participants in the control group (B=-0.94, SE=0.38, p=.013). This different was not apparent in the first 6 weeks

For peer review only - http://bmjopen.bmj.com/site/about/guidelines.xhtml

For peer review only

(B=0.22, SE=0.81, p=.78), but was apparent by week 12 (B=-3.06, SE=0.92, p=.001) with the difference between groups decreasing slightly by week 24 (B=-1.93, SE=1.12, p=.086) (see Figure 2A).

Depression

At baseline, just 14.8% (n=9) of participants showed mild depression and only 11.5% (n=7) showed moderate depression, with no participants showing severe depression. There was no evidence of a time by group interaction for depression (B=-0.49, SE=0.39, p=.21), with no difference evident at 6 weeks (B=-0.18, SE=0.81, p=.82), 12 weeks (B=-1.17, SE=0.94, p=.22) or 24 weeks (B=-1.28, SE=1.19, p=.28) (see Figure 2B).

Wellbeing

At baseline, 52.5% (n=32) of participants had poor wellbeing (<22), 16.4% (n=10) were below average (22–24), 16.4% (n=10) were average (25–26), 8.2% (n=5) were good (27–28) and 6.6% (n=4) were excellent (29– 35). There was a significant time by group interaction for wellbeing, with participants in the choir group showing a significantly greater increase in wellbeing than participants in the control group (B=1.25, SE=0.49, p=.011). This different was not apparent in the first 6 weeks (B=0.89, SE=0.98, p=.36) but was apparent by week 12 (B=3.58, SE=1.17, p=.002) and still present by week 24 (B=3.10, SE=1.50, p=.038) (see Figure 2C).

FIGURE 2 ABOUT HERE

Figure 2: Predictive margins of time by group interaction with 95% confidence intervals for (A) symptoms of anxiety, (B) symptoms of depression, (C) wellbeing. Control group is shown as a hard line, and experimental group as a dashed line.

Sensitivity analyses

We used sensitivity analyses to test whether participants in the choir group demonstrating anxiety and/or low wellbeing at baseline showed improvements over the 24 weeks compared to those in the control group.

Anxiety

As a first sensitivity analysis, we excluded participants with no evidence of anxiety at baseline (HADSA score<8) and re-ran analyses (n=27; control group=16, choir group=18). This confirmed a significant time by group interaction for anxiety, with participants in the choir group showing a significantly greater decrease in anxiety than participants in the

For peer review only - http://bmjopen.bmj.com/site/about/guidelines.xhtml

control group (B=-2.62, SE=0.58, p<.001). This different was not apparent in the first 6 weeks but was apparent by week 12 (B=-7.47, SE=1.28, p<.001) and was maintained at week 24 (B=-5.57, SE=1.64, p=.001).

These participants with anxiety also showed evidence of improvements in wellbeing, with a significant time by group interaction (B=1.93, SE=0.57, p=.001). As with full-sample wellbeing analyses, this difference was not apparent in the first 6 weeks (B=0.25, SE=1.08, p=0.81), but was apparent by week 12 (B=5.12, SE=1.27, p<.001) and was maintained at week 24 (B=4.32, SE=1.68, p=.01).

Wellbeing

As a second sensitivity analysis, we excluded participants with average or above average wellbeing at baseline (WEMWBS score>25) and re-ran analyses (n=42; control group=18, choir group=24). This confirmed a significant time by group interaction for wellbeing, with participants in the choir group showing a significantly greater improvement in wellbeing than participants in the control group (B=1.30, SE=0.51, p=.01). This different was not apparent in the first 6 weeks (B=1.45, SE=1.00, p=.15) but was apparent by week 12 (B=1.45, SE=1.16, p<.001), although it was slightly attenuated at week 24 (B=2.59, SE=1.52, p=.087).

These participants with low wellbeing also showed evidence of improvements in anxiety, with a significant time by group interaction (B=-1.22, SE=0.51, p=.017). As with full-sample anxiety analyses, this difference was not apparent in the first 6 weeks (B=0.95, SE=0.96, p=0.33), but was apparent by week 12 (B=-3.91, SE=1.10, p<.001), although it was slightly attenuated by week 24 (B=-2.34, SE=1.41, p=.097).

Singing continuation

As a third sensitivity analysis, we additionally factored in whether participants continued to sing beyond the first 12 weeks. But singing behaviours after the initial 12 weeks did not affect the significance of overall results nor significantly predict trajectories in anxiety, depression or wellbeing.

DISCUSSION

This study explored the effects of singing in a choir on mental health and wellbeing in people who care for somebody with cancer. Singing in a choir on a weekly basis was significantly associated with improvements both in anxiety and wellbeing, but not depression. These results were independent of demographic covariates, health-related covariates, previous or current engagement in musical activities, attitudes to singing and length of time caring. These

For peer review only - http://bmjopen.bmj.com/site/about/guidelines.xhtml

improvements in mental health and wellbeing were found amongst carers generally, as well as specifically carers with anxiety or below-average wellbeing. Differences between groups were not found within the first 6 weeks but were found by week 12 and were generally maintained at 3 month follow-up. 42% of carers involved in the choir group continued to sing in the follow-up period. However, continuing to sing beyond the initial 12 week period did not significantly predict wellbeing or anxiety at follow-up.

Our findings are in line with meta-analyses suggesting that anxiety rather than depression is the major challenge amongst carers [7]. In total, 56% of our participants showed symptoms of anxiety, with 35% specifically showing symptoms of moderate or severe anxiety. The finding that singing was associated with a greater decrease in anxiety echoes findings from previous studies of singing amongst people affected by cancer. In a study of 143 non-patients affected by cancer (comprising carers, staff and those who had been bereaved), singing reduced levels of anxiety but not depression using HADS over a 6-month period [21]. Further, our finding that benefits continued to be felt regardless of whether participants sung after the initial 12 weeks mirrors results from group drumming interventions for people with mild-moderate mental health conditions, which similarly found a maintenance of mental health benefits 3 months following the end of a 10-week programme [22].

A key consideration is how choir singing might reduce anxiety. A grounded theory study of the choirs involved in this study identified four key mechanisms [23]. First, the choirs provided emotional and uplifting experiences that participants experienced as a mind-body activity that supported their sense of identity. Second, the choirs provided social support; specifically an 'unspoken' group support that enabled the development of friendships, the perception of a caring network, and a sense of inspiration from the choir leader. Third, the choirs helped to build resilience amongst participants, developing coping skills, building confidence and leading to wider social and behavioural changes in individual's lives beyond the individual rehearsals themselves. Finally the choirs provided members with the chance to develop musical skills which enhanced their sense of self-esteem. In particular, the social support provided by the choirs may have been a key component for the carers in this study, as social support has been found to be a significant predictor of anxiety and quality of life in caregivers [1,16].

Strengths and limitations

This study had many strengths including its use of validated measures of mental health and wellbeing, its longitudinal tracking of effects of multiple time-points and its controlled design. We also considered factors such as additional psychological support being received by participants and deliberately only included participants who had not recently

For peer review only

For peer review only - http://bmjopen.bmj.com/site/about/guidelines.xhtml

had any change in their psychological support or medication. While the study was not randomised, this did have the advantage of mimicking real life choice in that participants had the option of selecting whether to enrol in the choir or not. Further, we then used a statistical approach that allowed us to model the effects of variables that could have affected exchangeability between groups on mental health trajectories across the 24 weeks. Notably, our groups were extremely well matched at baseline and results were not attenuated by the consideration of baseline confounders. However, our results were found in a relatively modest sample size, so future studies are required to ascertain whether results can be replicated in larger samples. Future studies could benefit from focusing more specifically on particular points in caregiving (such as around initial diagnosis or during palliative care). Such studies could also focus specifically on carers with known mental health diagnoses such as moderate anxiety, especially given the findings here that singing was of benefit both to those with and without baseline anxiety or low wellbeing.

Conclusions

In conclusion, this study builds on previous research showing the mental health benefits of singing for people with cancer by showing that weekly singing can also support anxiety and wellbeing in carers. Given that singing is a simple community activity free from the stigma that sometimes surrounds engagement with more formal support services, it could be promoted to support the mental health and wellbeing of cancer caregivers.

COMPETING INTERESTS

The funding for this study was provided by Tenovus Cancer Care who run the choirs used as the intervention in this study. However, no member of staff from Tenovus Cancer Care was involved in the specifics of the study design or in the data collection, analysis or interpretation of the data, the writing of the report, or the decision to submit the paper for publication.

FUNDING

This research was funded by Tenovus Cancer Care [SWU2016-01]. DF is funded by the Wellcome Trust [205407/Z/16/Z]. TW is funded by the Royal Marsden Cancer Charity. The research was facilitated by the infrastructure of the RM/ICR NIHR Biomedical Research Centre. The study team acknowledge the support of the National Institute of Health Research Clinical Research Network (NIHR CRN).

For peer review only - http://bmjopen.bmj.com/site/about/guidelines.xhtml

AUTHOR CONTRIBUTORSHIP STATEMENT

DF and TW designed the study. SF and KW collected the data. DF analysed the data and drafted the manuscript. All authors critically appraised the manuscript and approved it for publication.

DATA SHARING STATEMENT

Data from this study are available from the authors on request.

For peer review only

REFERENCES

1 Girgis A, Lambert S, Johnson C, *et al.* Physical, psychosocial, relationship, and economic burden of caring for people with cancer: a review. *J Oncol Pract* 2013;**9**:197–202. doi:10.1200/JOP.2012.000690

2 Northouse LL, Katapodi MC, Schafenacker AM, *et al.* The impact of caregiving on the psychological well-being of family caregivers and cancer patients. *Semin Oncol Nurs* 2012;**28**:236–45. doi:10.1016/j.soncn.2012.09.006

3 Stenberg U, Ruland CM, Miaskowski C. Review of the literature on the effects of caring for a patient with cancer. *Psychooncology* 2010;**19**:1013–25. doi:10.1002/pon.1670

4 Trevino KM, Prigerson HG, Maciejewski PK. Advanced cancer caregiving as a risk for major depressive episodes and generalized anxiety disorder. *Psychooncology* 2018;**27**:243–9. doi:10.1002/pon.4441

5 Hagedoorn M, Buunk BP, Kuijer RG, *et al.* Couples dealing with cancer: role and gender differences regarding psychological distress and quality of life. *Psychooncology* 2000;**9**:232–42.

6 Hodges LJ, Humphris GM, Macfarlane G. A meta-analytic investigation of the relationship between the psychological distress of cancer patients and their carers. *Soc Sci Med* 2005;**60**:1–12. doi:10.1016/j.socscimed.2004.04.018

7 Mitchell AJ, Ferguson DW, Gill J, *et al.* Depression and anxiety in long-term cancer survivors compared with spouses and healthy controls: a systematic review and meta-analysis. *The Lancet Oncology* 2013;**14**:721–32. doi:10.1016/S1470-2045(13)70244-4

8 Burridge LH, Barnett AG, Clavarino AM. The impact of perceived stage of cancer on carers' anxiety and depression during the patients' final year of life. *Psycho-Oncology*;**18**:615–23. doi:10.1002/pon.1435

9 Goren A, Gilloteau I, Lees M, *et al.* Quantifying the burden of informal caregiving for patients with cancer in Europe. *Support Care Cancer* 2014;**22**:1637–46. doi:10.1007/s00520-014-2122-6

10 Kim Y, Given BA. Quality of life of family caregivers of cancer survivors. *Cancer*;**112**:2556–68. doi:10.1002/cncr.23449

11 Frambes D, Given B, Lehto R, *et al.* Informal Caregivers of Cancer Patients: Review of Interventions, Care Activities, and Outcomes. *West J Nurs Res* 2018;**40**:1069–97. doi:10.1177/0193945917699364

12 Pitceathly C, Maguire P. The psychological impact of cancer on patients' partners and other key relatives: a review. *European Journal of Cancer* 2003;**39**:1517–24. doi:10.1016/S0959-8049(03)00309-5

13 Northouse LL, Katapodi MC, Song L, *et al.* Interventions with family caregivers of cancer patients: meta-analysis of randomized trials. *CA Cancer J Clin* 2010;**60**:317–39. doi:10.3322/caac.20081

14 Badr H, Krebs P. A systematic review and meta-analysis of psychosocial interventions for couples coping with cancer. *Psychooncology* 2013;**22**:1688–704. doi:10.1002/pon.3200

15 Aoun SM, Kristjanson LJ, Currow DC, *et al.* Caregiving for the terminally ill: at what cost? *Palliat Med* 2005;**19**:551–5. doi:10.1191/0269216305pm1053oa

16 Mellon S, Northouse LL, Weiss LK. A population-based study of the quality of life of cancer survivors and their family caregivers. *Cancer Nurs* 2006;**29**:120–31; quiz 132–3.

17 Fancourt D, Warran K. Singing for cancer: implications from psychoneuroimmunology. In: *Advances in Interdisciplinary Research in Singing*. Routledge 2018.

18 Fancourt D, Williamon A, Carvalho LA, *et al.* Singing modulates mood, stress, cortisol, cytokine and neuropeptide activity in cancer patients and carers. *ecancermedicalscience* 2016;**10**. doi:10.3332/ecancer.2016.631

19 Morris JR, Dow R. A model for the continuous quality improvement of the Sing With Us choir leaders. *Journal of Arts & Communities* 2014;**6**:133–144.

20 Tennant R, Hiller L, Fishwick R, *et al.* The Warwick-Edinburgh Mental Well-being Scale (WEMWBS): development and UK validation. *Health and Quality of Life Outcomes* 2007;**5**:63. doi:10.1186/1477-7525-5-63

21 Reagon C, Gale N, Dow R, *et al.* Choir singing and health status in people affected by cancer. *Eur J Cancer Care (Engl)* 2017;**26**. doi:10.1111/ecc.12568

22 Fancourt D, Perkins R, Ascenso S, *et al.* Effects of Group Drumming Interventions on Anxiety, Depression, Social Resilience and Inflammatory Immune Response among Mental Health Service Users. *PLOS ONE* 2016;**11**:e0151136. doi:10.1371/journal.pone.0151136

23 Warran K, Fancourt D, Wiseman T. How does the process of group singing impact upon people affected by cancer? A grounded theory study. *BMJ Open*

Figure 1: CONSORT diagram of participants

Figure 2: Predictive margins of time by group interaction with 95% confidence intervals for (A) symptoms of anxiety, (B) symptoms of depression, (C) wellbeing. Control group is shown as a hard line, and experimental group as a dashed line.

For peer review only

For peer review only - http://bmjopen.bmj.com/site/about/guidelines.xhtml

Figure 1: CONSORT diagram of participants

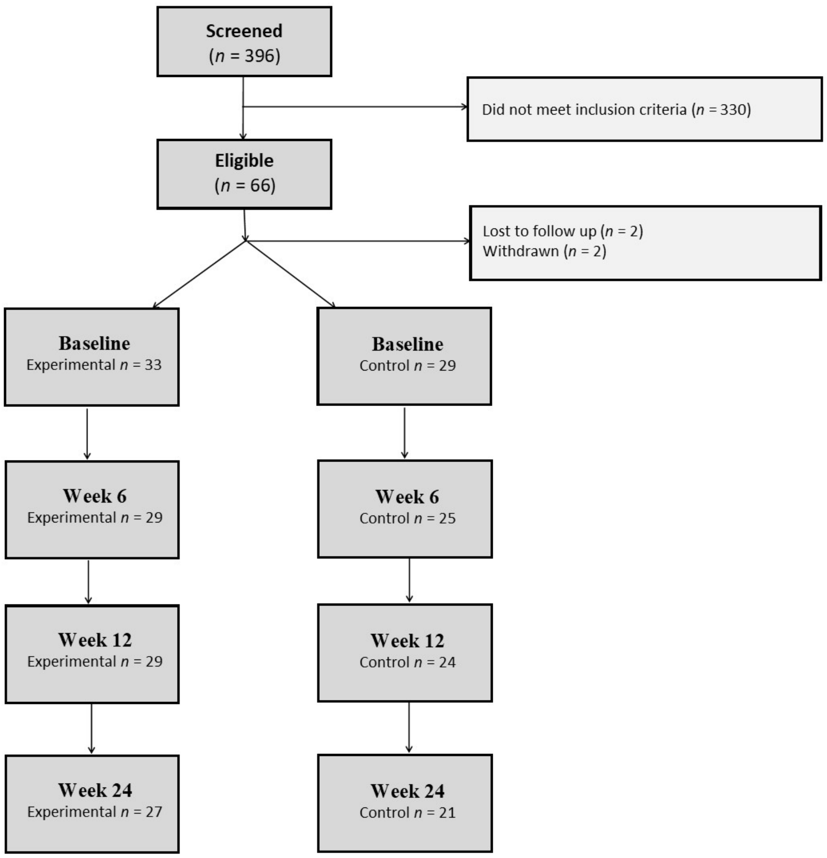

Figure 1

212x194mm (150 x 150 DPI)

Figure 2: Predictive margins of time by group interaction with 95% confidence intervals for (A) symptoms of anxiety, (B) symptoms of depression, (C) wellbeing. Control group is shown as a hard line, and experimental group as a dashed line.

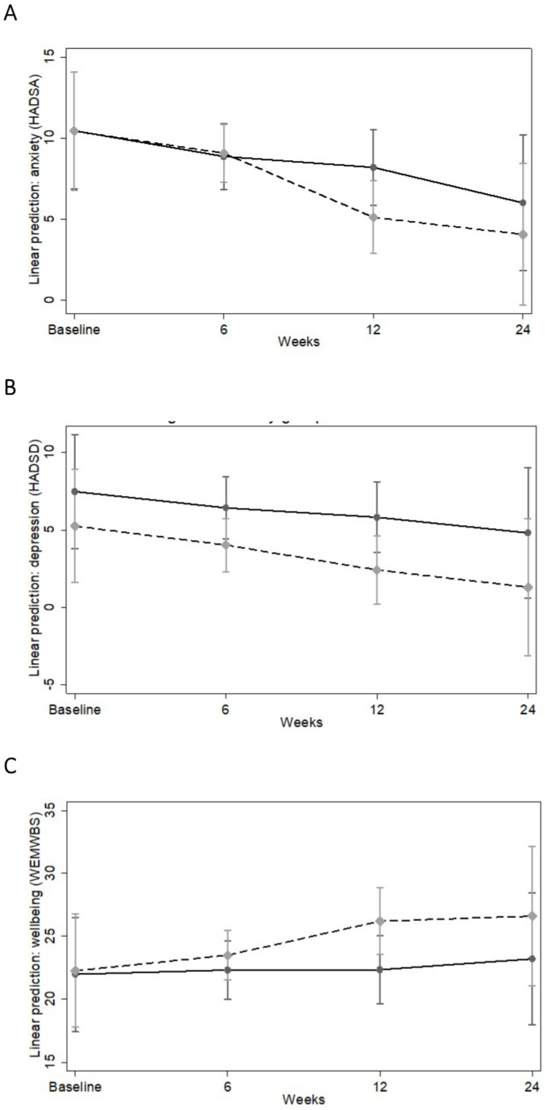

Figure 2

215x271mm (150 x 150 DPI)

BMJ Open

Psychosocial singing interventions for the mental health and wellbeing of family carers of cancer patients: results from a longitudinal controlled study

Journal:	*BMJ Open*
Manuscript ID	bmjopen-2018-026995.R1
Article Type:	Research
Date Submitted by the Author:	02-Apr-2019
Complete List of Authors:	Fancourt, Daisy; University College London Research Department of Epidemiology and Public Health, Department of Behavioural Science and Health<br>Warran, Katey; The University of Edinburgh, Edinburgh College of Art<br>Finn, Saoirse; Royal College of Music, Centre for Performance Science<br>Wiseman, Theresa; The Royal Marsden NHS Foundation Trust, ; University of Southampton, Faculty of Health Sciences
**Primary Subject Heading**:	Health services research
Secondary Subject Heading:	Complementary medicine, Mental health, Patient-centred medicine
Keywords:	Anxiety disorders < PSYCHIATRY, MENTAL HEALTH, Depression & mood disorders < PSYCHIATRY, SOCIAL MEDICINE, ONCOLOGY

SCHOLARONE™
Manuscripts

Psychosocial singing interventions for the mental health and wellbeing of family carers of cancer patients: results from a longitudinal controlled study

SHORT RUNNING TITLE: Psychosocial singing interventions for the mental health of cancer carers

Fancourt D[1]*, Warran K[2], Finn S[2], Wiseman T[3,4]

[1] Department of Behavioural Science and Health, University College London, UK

[2] Centre for Performance Science, Royal College of Music, London, UK

[3] The Royal Marsden NHS Foundation Trust, London, UK

[4] Faculty of Health Sciences, University of Southampton, Southampton, UK

* Corresponding author: Dr Daisy Fancourt, University College London, 1-19 Torrington Place, London WC1E 7HB, d.fancourt@ucl.ac.uk

Word count: 2099

For peer review only - http://bmjopen.bmj.com/site/about/guidelines.xhtml

ABSTRACT

Objective. The mental health challenges facing people who care for somebody with cancer are well documented. While many support interventions focus on provision of information or cognitive behavioural therapy, the literature suggests that psychosocial interventions could also be of value, especially given the low social support frequently reported by carers. Singing is a psychosocial activity shown to improve social support, increase positive emotions and reduce fatigue and stress. This study explored the effects of weekly group singing on anxiety, depression and wellbeing in cancer carers over a six-month period.

Design. A multi-site non-randomised longitudinal controlled study

Setting. The Royal Marsden NHS Trust in Greater London.

Participants. 62 adults who currently care for a spouse, relative or close friend with cancer who had not recently started any medication or psychological therapy.

Interventions. On enrolment, participants selected to join a weekly community choir for 12 weeks (n=33) or continue with life as usual (n=29).

Outcome measures. The primary outcome was mental health using the Hospital Anxiety and Depression Scale. Secondary outcomes were wellbeing using the Warwick Edinburgh Mental Wellbeing Scale. Using linear mixed effects models, we compared the change in mental health and wellbeing over time between the two groups while adjusting for confounding variables including demographics, health-related variables, musical engagement and length of time caring.

Results. Participants in the choir group showed a significantly greater decrease in anxiety over time than participants in the control group (B=-0.94, SE=0.38, p=.013), and a significantly greater increase in wellbeing (B=1.25, SE=0.49, p=.011). No changes were found for depression. Sub-group analyses showed carers with anxiety or below-average wellbeing were most likely to benefit.

Conclusions. This study builds on previous research showing the mental health benefits of singing for people with cancer by showing that weekly singing can also support anxiety and wellbeing in cancer carers.

KEYWORDS

Cancer, oncology, carers, anxiety, depression, wellbeing, singing, psychosocial, social support

For peer review only - http://bmjopen.bmj.com/site/about/guidelines.xhtml

ARTICLE SUMMARY

Strengths and limitations of this study

- We used random-intercept random-slope linear mixed effects models which allowed us to compare different rates of change in mental health and wellbeing over time amongst carers who did and didn't take part in a weekly choir over 24 weeks.
- Our results were found independent of identified confounding factors including demographics, health-related variables, musical engagement and length of time caring.
- This study had many strengths including its longitudinal tracking of effects of multiple time-points and its controlled design.
- Our groups were statistically well matched at baseline and results were not attenuated by the consideration of baseline confounders.
- However, our results were found in a relatively modest sample size, so future studies are required to ascertain whether results can be replicated in larger samples.
- Further, our study was not randomised so we cannot assume full exchangeability between groups.

For peer review only

For peer review only - http://bmjopen.bmj.com/site/about/guidelines.xhtml

INTRODUCTION

Family members and close friends, particularly spouses, are often primary caregivers for individuals affected by cancer, playing a vital role in their care process. Yet, well documented across the literature are the psychological, physical, social and economic challenges faced by informal cancer carers [1,2]. In relation to *mental health*, carers have repeatedly been found to have greater anxiety and depression levels than general population controls [3,4]. Indeed, psychological distress in carers correlates with the distress experienced by patients and has been found through meta-analysis to be no different in size [5,6]. Prevalence rates of depression are around 26% in both patients and spouses and the prevalence of anxiety is around 28% in patients and 40% in spouses [7]. In particular, anxiety has been found to increase when caring for somebody with advanced cancer, exceeding depression in carers [8]. This has led to suggestions that anxiety, rather than depression, is the largest psychological problem in carers and calls for interventions that can tackle it [7].

In addition to *mental health* challenges, caring for somebody with cancer has also been found to affect *wellbeing*. A Europe-wide survey has found mental as well as physical health-related quality of life to be significantly lower in carers than non-caregivers [9]. Female carers in particular report lower quality of life than women who are not carers [5], and quality of life for carers of both genders has been found to vary across the illness trajectory [10]. Given that wellbeing is associated with mental health and also related factors such as resilience and coping, there is a need too for interventions that can support wellbeing in carers.

Many interventions for carers focus on the provision of information designed to directly support carers in their roles, or involve cognitive behavioural therapy to support and enhance coping [11,12]. A meta-analysis of therapeutic counselling, skills training and psychoeducational interventions for caregivers of cancer patients found evidence of reductions in caregiving burden, enhanced coping strategies, self-efficacy, physical functioning, anxiety, family relations and social functioning [13]. Interventions were identified as being more effective if caregivers attended alone (i.e. without the person they cared for) and if the interventions were delivered in a group setting with other carers. However, there has also been increasing interest in the role of psychosocial interventions for supporting mental health and wellbeing in carers (e.g. [14]. Such interest has been encouraged by research suggesting that, in addition to specific needs relating to their caregiving, carers frequently have unmet personal needs and low social support themselves [15]. In a population-based study of family carers of cancer survivors, social support was one of the

For peer review only - http://bmjopen.bmj.com/site/about/guidelines.xhtml

For peer review only

strongest predictors of quality of life [16]. Carers with less social support also have a higher risk of developing depression and anxiety [1]. Yet, caregivers often feel that they receive less support in comparison to how much patients feel that they receive [16].

Singing is a promising multimodal psychosocial intervention that has been used to support mental health and wellbeing in diverse populations, including amongst cancer patients [17]. In cancer caregivers, singing has been shown to increase perceived social support, enhance short-term positive emotions, reduce fatigue, promote relaxation and also reduce levels of stress hormones and mood-related inflammation over a single one hour singing session [18]. However, to date there is little research into whether singing can specifically reduce anxiety and depression and enhance wellbeing in cancer caregivers, and the effects of singing over longer periods of time. Whilst a few studies have identified benefits of singing for other groups of carers, such as carers of people with dementia, these studies have typically been very small in sample size (e.g. 4-7 participants completing data collection), followed participants over relatively short time frames (e.g. just 6 weeks), and employed little quantitative data [19–22]. Consequently, this study built on the theoretical rationale for the benefits of psychosocial interventions for cancer carers and the preliminary data showing the benefits of singing specifically for carer populations and explored the effects of weekly singing on anxiety, depression and wellbeing in carers of somebody affected by cancer over a six-month period.

MATERIALS AND METHODS

Participants and procedure

This study was a multi-site non-randomised longitudinal controlled study. To be eligible, participants had to self-identify as currently caring for a spouse, relative or close friend with cancer. We excluded members of hospital staff in order to differentiate between formal and informal care. Participants were excluded if they were under the age of 18, if they were already engaged in a weekly group choir, if they had started a formal course of psychological therapy in the last month or were scheduled to start one in the next 12 weeks, if they had started any new medication for anxiety or depression in the last month, or if their level of English was insufficient to complete the questionnaires required. Participants were recruited through NHS hospital trusts in Greater London, carer support groups, community and charity events and social media. A total of 396 were screened for enrolment, 66 were eligible and consented to take part, and 62 participants (29 control and 33 experimental) enrolled in the study (see Figure 1 for CONSORT diagram

For peer review only - http://bmjopen.bmj.com/site/about/guidelines.xhtml

of participants). The study received ethical approval from the National Research Ethics Service [16/LO/0579] and all participants provided informed consent.

FIGURE 1 ABOUT HERE

Figure 1: CONSORT diagram of participants

On enrolment, participants were given the option of joining a weekly choir for 12 weeks. This length of study was selected from pilot data collected on singing for patients affected by cancer who were involved in uncontrolled studies in Wales, which suggested that improvements in mental health were found 12 weeks following joining, with further improvements by 24 weeks [23]. Participants who did not select to join a choir formed the control group and continued with life as usual. Participants who did select to join a choir took part in a 90-minute session each week led by a professional choir leader, of which 60 minutes was dedicated to singing (including warm-up exercises, learning new songs and singing familiar songs) and 30 minutes to socialising. The repertoire focused on popular songs arranged specifically for the choirs with backing tracks. All songs were learnt by ear without participants need to read music. These choirs were led by Tenovus Cancer Care, specifically for people affected in some way by cancer, and the format of the sessions has been described in previous studies [24]. Both choirs used in this study were established specifically for the purposes of collecting data. Following 12 weeks in the choir, participants were given the option to continue with weekly choir sessions if they wished, with 19 participants (58%) choosing to leave and the remaining 42% choosing to continue attending. Those who continued attending attended an average of 10 sessions out of 12, with 57% of them attending all 12. All participants were asked to complete questionnaires at baseline, 6 and 12 weeks as well as at 3-month follow-up (24 weeks).

Patient and Public Involvement

The research questions and outcome measures were selected with the support of a Patient and Public Involvement (PPI) group who were participants in the Tenovus Cancer Choirs in Wales. These participants also approved the design of the study. As this was the first study of the choirs at the Royal Marsden Hospital sites (or indeed in England) and there was therefore not data about the response from individuals in the community or the hospitals to the choirs, it was proposed by our PPI group that the study could be run as a longitudinal controlled study rather than a randomised controlled trial (RCT). As such, any carers would have the option of joining immediately rather than becoming part of

For peer review only - http://bmjopen.bmj.com/site/about/guidelines.xhtml

a wait-list control. Whilst this has obvious methodological limitations, it was felt to be an important precursor to an RCT that would enable an assessment of the reception of the choirs in these new locations alongside measurements of outcome data. Participants in the study also supported recruitment efforts by helping to identify further participants. Dissemination plans include presentations back to members of all of the Tenovus Cancer Choirs in Wales and England.

Measures

Our primary outcomes were anxiety and depression, measured using the Hospital Anxiety and Depression Scale (HADS), which contains two independent scales: one for anxiety and one for depression ranging from 0-21 for each construct with higher scores indicating poorer mental health (Bjelland, Dahl, Haug, & Neckelmann, 2002). When using this measure, scores of 0-7 are considered as non-indicative of anxiety or depression, while scores of 8-10 present mild cases, 11-14 present moderate cases, and 15-21 present severe cases [25].

Our secondary outcome was wellbeing, measured using the Warwick Edinburgh Mental Wellbeing Scale (WEMWBS) short form, which encompasses both hedonic and eudemonic wellbeing [26]. The scale is scored from 7-35 with higher scores representing higher levels of wellbeing. As recommended in validations, the raw scores were logit transformed prior to analysis. The New Economics Foundation suggests five levels of wellbeing based on quintile analyses of data in the UK Understanding Society Survey, 2009: poor (<22), below average (22–24), average (25–26), good (27–28) and excellent (29– 35).

Additionally, we collected data on age, gender, income (<£16,000, £16,000-£30,000, £31,000-£60,000, £61,000-£90,000, >£90,000), employment status (unemployed, voluntary/temporary work, part-time work, full-time work, retired), whether participants were receiving psychological therapy, whether participants had any other health condition, whether participants had previously sung in a choir, how confident participants felt about singing (from 1-not at all to 5-very), whether participants had attended musical concerts or performances in the past year, whether they had taken part in any other music activity in the past year, and how long participants had been in a caring role for.

Statistics

To compare the choir vs the control group at baseline, we used one-way analyses of variance, and Fisher's exact tests. To explore changes in mental health and wellbeing over time and between groups, we used linear mixed effects

For peer review only - http://bmjopen.bmj.com/site/about/guidelines.xhtml

models (LMMs). LMMs allow for natural heterogeneity amongst participants and, unlike some other repeated measures analysis models such as repeated measures analyses of variance which deal with missing data through list-wise deletion, linear mixed effects models make full use of the dataset. Our participants provided data an average of 3.4 times across the four time points, providing 180 data points. Our models used a random intercept and random slope, with an unstructured covariance matrix of the random effects. Time was modelled first as both a continuous variable to identify an overall time*group relationship (using a linear model as we found no evidence to support the use of a quadratic model), and then time was modelled as four separate time points to identify where specific changes occurred. We tested quadratic models but found no evidence to support their usage over a linear model. All models demonstrated normality of residuals and no evidence of heteroscedasticity.

We built up our final model through considering nested models of relevant covariates. Model 1 was unadjusted apart from baseline mental health, model 2 adjusted for demographic variables (age, gender, income and employment status), model 3 additionally adjusted for health-related variables (undergoing psychological therapy, and any other health conditions), model 4 additionally adjusted for cultural engagement and attitudes to singing (past experiences singing in a choir, confidence in singing, attendance at concerts or performances in the past year, and engagement in any other musical activity in the past year), and model 5 additionally adjusted for length of time caring for somebody with cancer. We used the log-likelihood ratio, Akaike's information criterion and Bayesian information criterion to confirm best fit of model 5. We calculated margins of response from the fully-adjusted model and created profile plots to illustrate the time*group interactions. As three outcomes were considered, a Bonferroni's alpha of 0.05/3=0.017 can be assumed. Standardised residuals were generated to confirm the assumption of normality.

In order to ascertain whether those with poorer mental health and wellbeing were more or less likely to benefit from engagement, sub-group analyses excluded those with no evident mental health problems at baseline. Additionally, given participants had been given the option of whether to continue with weekly singing following the first 12 weeks, we statistically compared differences in demographics and mental health amongst those who did and did not continue and also ran sensitivity analyses additionally adjusting for singing patterns after the initial 12 weeks to see if these affected outcomes at 24 weeks. All results shown are fully-adjusted models (i.e. Model 5) and all analyses were conducted using Stata v14.

For peer review only - http://bmjopen.bmj.com/site/about/guidelines.xhtml

RESULTS

Demographics

Participants were well matched at baseline on all demographic variables including mental health and wellbeing. The only significant difference between groups was in confidence in singing, with the control group showing lower overall confidence than the singing group (see Table 1).

Table 1: Baseline demographics of participants

	Control (n=29)	Choir (n=33)	P value
Age, mean (SD)	51 (15)	58 (14)	0.075 [a]
Sex, % female	72.4%	81.8%	0.54 [b]
Income, %			0.076 [b]
<£16,000	12.0%	20.7%	
£16,000-£30,000	24.0%	41.4%	
£31,000-£60,000	32.0%	24.1%	
£61,000-£90,000	32.0%	6.9%	
>£91,000	0%	6.9%	
Employment status, %			0.22 [b]
Unemployed	13.8%	3.2%	
Voluntary	0%	6.5%	
Part-time work	20.7%	29.0%	
Full-time work	37.9%	22.6%	
Retired	27.6%	38.7%	
Currently having therapy, %	10.3%	24.2%	0.19 [b]
Health condition, %	72.4%	60.6%	0.42 [b]
Previously sung in a choir, %	65.5%	48.5%	0.21 [b]
Confidence in singing, %			**0.040** [c]
1 – not at all	50%	21.2%	
2	21.4%	12.1%	
3	21.4%	36.4%	
4	3.6%	21.2%	
5 – very	3.6%	9.1%	
Attended a concert or performance in the past month, %	32.1%	51.5%	0.19 [b]
Took part in a music activity in the past month, %	18.5%	36.4%	0.16 [b]
Length of time being a carer, %			0.45 [c]
0-3 months	0%	9.7%	
3-12 months	17.9%	19.4%	
1-3 years	50%	35.5%	
3-5 years	14.3%	22.6%	
5+ years	17.9%	12.9%	
Anxiety, mean (SD)	8.04 (4.71)	8.97 (4.19)	0.42 [a]
Depression, mean (SD)	5.43 (4.49)	4.88 (3.08)	0.57 [a]
Wellbeing, mean (SD)	23.15 (4.36)	21.71 (3.70)	0.17 [a]

[a] one-way ANOVA; [b] Fisher's exact test

Anxiety

At baseline, 44.3% (n=27) of participants showed no anxiety (HADS Anxiety (HADSA) score 0-7), 21.3% (n=13) showed mild anxiety (HADSA score 8-10), 23.0% (n=14) showed moderate anxiety (HADSA score 11-14), and

For peer review only

For peer review only - http://bmjopen.bmj.com/site/about/guidelines.xhtml

11.5% (n=7) showed severe anxiety (HADSA score 15-21). The linear mixed effects model with time as a continuous variable showed that there was a significant time by group interaction, with participants in the choir group showing a significantly greater decrease in anxiety than participants in the control group (B=-0.94, SE=0.38, p=.013). When modelling time as four separate time points, this difference was not apparent in the first 6 weeks (B=0.22, SE=0.81, p=.78), but was apparent by week 12 (B=-3.06, SE=0.92, p=.001), with the difference between groups decreasing slightly by week 24 (B=-1.93, SE=1.12, p=.086) (see Figure 2A). In the choir group, this equated to 17.5% decrease in anxiety symptoms across the first 12 weeks, which had slightly decreased to an 11.9% decrease in anxiety symptoms by 24 weeks.

Depression

At baseline, just 14.8% (n=9) of participants showed mild depression and only 11.5% (n=7) showed moderate depression, with no participants showing severe depression. There was no evidence of a time by group interaction for depression with time as a continuous variable (B=-0.49, SE=0.39, p=.21). When modelling time as four separate time points, there was similarly no difference evident at 6 weeks (B=-0.18, SE=0.81, p=.82), 12 weeks (B=-1.17, SE=0.94, p=.22), or 24 weeks (B=-1.28, SE=1.19, p=.28) (see Figure 2B).

Wellbeing

At baseline, 52.5% (n=32) of participants had poor wellbeing (<22), 16.4% (n=10) were below average (22–24), 16.4% (n=10) were average (25–26), 8.2% (n=5) were good (27–28) and 6.6% (n=4) were excellent (29– 35). There was a significant time by group interaction for wellbeing when modelling time as a continuous variable, with participants in the choir group showing a significantly greater increase in wellbeing than participants in the control group (B=1.25, SE=0.49, p=.011). When modelling time as four separate time points, this difference was not apparent in the first 6 weeks (B=0.89, SE=0.98, p=.36), but was apparent by week 12 (B=3.58, SE=1.17, p=.002). and still present by week 24 (B=3.10, SE=1.50, p=.038) (see Figure 2C). This equated to a 12.6% increase in wellbeing in the choir group across the first 12 weeks, which had slightly decreased to a 10.5% increase by week 24.

FIGURE 2 ABOUT HERE

Figure 2: Predictive margins of time by group interaction with 95% confidence intervals for (A) symptoms of anxiety, (B) symptoms of depression, (C) wellbeing. Control group is shown as a hard line, and experimental group as a dashed line. The model is adjusted for demographic factors, health-related factors, cultural engagement and attitudes to singing, and length of time caring for somebody with cancer.

For peer review only - http://bmjopen.bmj.com/site/about/guidelines.xhtml

For peer review only

Sub-group and sensitivity analyses

We used sub-group analyses to test whether participants in the choir group demonstrating anxiety and/or low wellbeing at baseline showed improvements over the 24 weeks compared to those in the control group.

Anxiety

As a first sub-group analysis, we excluded participants with no evidence of anxiety at baseline (HADSA score<8) and re-ran analyses (n=27; control group=16, choir group=18). This confirmed a significant time by group interaction for anxiety, with participants in the choir group showing a significantly greater decrease in anxiety than participants in the control group (B=-2.62, SE=0.58, p<.001). This difference was not apparent in the first 6 weeks, was was apparent by week 12 (B=-7.47, SE=1.28, p<.001), and was maintained at week 24 (B=-5.57, SE=1.64, p=.001).

These participants with anxiety also showed evidence of improvements in wellbeing, with a significant time by group interaction (B=1.93, SE=0.57, p=.001). As with full-sample wellbeing analyses, this difference was not apparent in the first 6 weeks (B=0.25, SE=1.08, p=0.81), but was apparent by week 12 (B=5.12, SE=1.27, p<.001), and was maintained at week 24 (B=4.32, SE=1.68, p=.01).

Wellbeing

As a second sub-group analysis, we excluded participants with average or above average wellbeing at baseline (WEMWBS score>25) and re-ran analyses (n=42; control group=18, choir group=24). This confirmed a significant time by group interaction for wellbeing, with participants in the choir group showing a significantly greater improvement in wellbeing than participants in the control group (B=1.30, SE=0.51, p=.01). This difference was not apparent in the first 6 weeks (B=1.45, SE=1.00, p=.15), but was apparent by week 12 (B=1.45, SE=1.16, p<.001), although it was slightly attenuated at week 24 (B=2.59, SE=1.52, p=.087).

These participants with low wellbeing also showed evidence of improvements in anxiety, with a significant time by group interaction (B=-1.22, SE=0.51, p=.017). As with full-sample anxiety analyses, this difference was not apparent in the first 6 weeks (B=0.95, SE=0.96, p=0.33), but was apparent by week 12 (B=-3.91, SE=1.10, p<.001), although it was slightly attenuated by week 24 (B=-2.34, SE=1.41, p=.097).

Singing continuation

We compared differences between those who did and did not continue with singing beyond the first 12 weeks. There were no demographic differences between groups or differences in mental health between those who continued with

For peer review only

For peer review only - http://bmjopen.bmj.com/site/about/guidelines.xhtml

the singing following week 12. As a sensitivity analysis, we additionally factored in whether participants continued to sing beyond the first 12 weeks on our primary and secondary outcomes. Singing behaviours after the initial 12 weeks were found to not affect the significance of overall results nor significantly predict trajectories in anxiety, depression or wellbeing.

DISCUSSION

This study explored the effects of singing in a choir on mental health and wellbeing in people who care for somebody with cancer. Singing in a choir on a weekly basis was significantly associated with improvements in anxiety and wellbeing, but not depression. These results were independent of demographic covariates, health-related covariates, previous or current engagement in musical activities, attitudes to singing and length of time caring. These improvements in mental health and wellbeing were found amongst carers generally, as well as specifically carers with anxiety or below-average wellbeing. Differences between groups were not found within the first 6 weeks but were found by week 12 and were generally maintained at the 3-month follow-up. 42% of carers involved in the choir group continued to sing in the follow-up period. However, continuing to sing beyond the initial 12 week period did not significantly predict wellbeing or anxiety at follow-up.

Our findings are in line with meta-analyses suggesting that anxiety rather than depression is the major challenge amongst cancer carers [7]. In total, 56% of our participants showed symptoms of anxiety, with 35% specifically showing symptoms of moderate or severe anxiety. The finding that singing was associated with a greater decrease in anxiety echoes findings from previous studies of singing amongst people affected by cancer. In a study of 143 non-patients affected by cancer (comprising carers, staff and those who had been bereaved), singing reduced levels of anxiety but not depression using HADS over a 6-month period [23]. Further, our finding that benefits continued to be felt regardless of whether participants sung after the initial 12 weeks mirrors results from group drumming interventions for people with mild-moderate mental health conditions, which similarly found a maintenance of mental health benefits 3 months following the end of a 10-week programme [27].

A key consideration is how choir singing might reduce anxiety. A separate grounded theory study of the choirs involved in this study identified four key mechanisms [28]. First, the choirs provided emotional and uplifting experiences that participants experienced as a mind-body activity that supported their sense of identity. Second, the choirs provided social support; specifically an 'unspoken' group support that enabled the development of friendships,

For peer review only - http://bmjopen.bmj.com/site/about/guidelines.xhtml

the perception of a caring network, and a sense of inspiration from the choir leader. Third, the choirs helped to build resilience amongst participants, developing coping skills, building confidence and leading to wider social and behavioural changes in individual's lives beyond the individual rehearsals themselves. Finally, the choirs provided members with the chance to develop musical skills which enhanced their sense of self-esteem. In particular, the social support provided by the choirs may have been a key component for the carers in this study, as social support has been found to be a significant predictor of anxiety and quality of life in caregivers [1,16].

Strengths and limitations

This study had many strengths including its longitudinal tracking of effects of multiple time-points and its controlled design. We also considered factors such as additional psychological support being received by participants and deliberately only included participants who had not recently had any change in their psychological support or medication. While the study was not randomised, this did have the advantage of mimicking real life choice in that participants had the option of selecting whether to enrol in the choir or not. Further, we then used a statistical approach that allowed us to model the effects of variables that could have affected exchangeability between groups on mental health trajectories across the 24 weeks. Notably, our groups were extremely well matched statistically at baseline and results were not attenuated by the consideration of baseline confounders. However, our results were found in a relatively modest sample size, so future studies are required to ascertain whether results can be replicated in larger samples. Future studies could benefit from focusing more specifically on particular points in caregiving (such as around initial diagnosis or during palliative care). Such studies could also focus specifically on carers with known mental health diagnoses such as moderate anxiety, especially given the findings here that singing was of benefit both to those with and without baseline anxiety or low wellbeing. Finally, this study focused exclusively on carers of somebody with cancer. However, whether the longitudinal benefits of group singing found here are replicated amongst other carer groups remains to be explored.

Conclusions

In conclusion, this study builds on previous research showing the mental health benefits of singing for people affected by cancer by showing that weekly singing can also support anxiety and wellbeing in carers. Given that singing is a simple community activity free from the stigma that sometimes surrounds engagement with more formal support services, it could be promoted to support the mental health and wellbeing of cancer caregivers.

For peer review only - http://bmjopen.bmj.com/site/about/guidelines.xhtml

COMPETING INTERESTS

The funding for this study was provided by Tenovus Cancer Care who run the choirs used as the intervention in this study. However, no member of staff from Tenovus Cancer Care was involved in the specifics of the study design or in the data collection, analysis or interpretation of the data, the writing of the report, or the decision to submit the paper for publication.

FUNDING

This research was funded by Tenovus Cancer Care [SWU2016-01]. DF is funded by the Wellcome Trust [205407/Z/16/Z]. TW is funded by the Royal Marsden Cancer Charity. The research was facilitated by the infrastructure of the RM/ICR NIHR Biomedical Research Centre. The study team acknowledge the support of the National Institute of Health Research Clinical Research Network (NIHR CRN).

AUTHOR CONTRIBUTORSHIP STATEMENT

DF and TW designed the study. SF and KW collected the data. DF analysed the data and drafted the manuscript. All authors critically appraised the manuscript and approved it for publication.

DATA SHARING STATEMENT

Data from this study are available from the authors on request.

REFERENCES

1 Girgis A, Lambert S, Johnson C, *et al.* Physical, psychosocial, relationship, and economic burden of caring for people with cancer: a review. *J Oncol Pract* 2013;**9**:197–202. doi:10.1200/JOP.2012.000690

2 Northouse LL, Katapodi MC, Schafenacker AM, *et al.* The impact of caregiving on the psychological well-being of family caregivers and cancer patients. *Semin Oncol Nurs* 2012;**28**:236–45. doi:10.1016/j.soncn.2012.09.006

3 Stenberg U, Ruland CM, Miaskowski C. Review of the literature on the effects of caring for a patient with cancer. *Psychooncology* 2010;**19**:1013–25. doi:10.1002/pon.1670

For peer review only - http://bmjopen.bmj.com/site/about/guidelines.xhtml

4 Trevino KM, Prigerson HG, Maciejewski PK. Advanced cancer caregiving as a risk for major depressive episodes and generalized anxiety disorder. *Psychooncology* 2018;**27**:243–9. doi:10.1002/pon.4441

5 Hagedoorn M, Buunk BP, Kuijer RG, *et al.* Couples dealing with cancer: role and gender differences regarding psychological distress and quality of life. *Psychooncology* 2000;**9**:232–42.

6 Hodges LJ, Humphris GM, Macfarlane G. A meta-analytic investigation of the relationship between the psychological distress of cancer patients and their carers. *Soc Sci Med* 2005;**60**:1–12. doi:10.1016/j.socscimed.2004.04.018

7 Mitchell AJ, Ferguson DW, Gill J, *et al.* Depression and anxiety in long-term cancer survivors compared with spouses and healthy controls: a systematic review and meta-analysis. *The Lancet Oncology* 2013;**14**:721–32. doi:10.1016/S1470-2045(13)70244-4

8 Burridge LH, Barnett AG, Clavarino AM. The impact of perceived stage of cancer on carers' anxiety and depression during the patients' final year of life. *Psycho-Oncology*;**18**:615–23. doi:10.1002/pon.1435

9 Goren A, Gilloteau I, Lees M, *et al.* Quantifying the burden of informal caregiving for patients with cancer in Europe. *Support Care Cancer* 2014;**22**:1637–46. doi:10.1007/s00520-014-2122-6

10 Kim Y, Given BA. Quality of life of family caregivers of cancer survivors. *Cancer*;**112**:2556–68. doi:10.1002/cncr.23449

11 Frambes D, Given B, Lehto R, *et al.* Informal Caregivers of Cancer Patients: Review of Interventions, Care Activities, and Outcomes. *West J Nurs Res* 2018;**40**:1069–97. doi:10.1177/0193945917699364

12 Pitceathly C, Maguire P. The psychological impact of cancer on patients' partners and other key relatives: a review. *European Journal of Cancer* 2003;**39**:1517–24. doi:10.1016/S0959-8049(03)00309-5

13 Northouse LL, Katapodi MC, Song L, *et al.* Interventions with family caregivers of cancer patients: meta-analysis of randomized trials. *CA Cancer J Clin* 2010;**60**:317–39. doi:10.3322/caac.20081

14 Badr H, Krebs P. A systematic review and meta-analysis of psychosocial interventions for couples coping with cancer. *Psychooncology* 2013;**22**:1688–704. doi:10.1002/pon.3200

15 Aoun SM, Kristjanson LJ, Currow DC, *et al.* Caregiving for the terminally ill: at what cost? *Palliat Med* 2005;**19**:551–5. doi:10.1191/0269216305pm1053oa

16 Mellon S, Northouse LL, Weiss LK. A population-based study of the quality of life of cancer survivors and their family caregivers. *Cancer Nurs* 2006;**29**:120–31; quiz 132–3.

17 Fancourt D, Warran K. Singing for cancer: implications from psychoneuroimmunology. In: *Advances in Interdisciplinary Research in Singing*. Routledge 2018.

18 Fancourt D, Williamon A, Carvalho LA, *et al.* Singing modulates mood, stress, cortisol, cytokine and neuropeptide activity in cancer patients and carers. *ecancermedicalscience* 2016;**10**. doi:10.3332/ecancer.2016.631

19 Camic PM, Williams CM, Meeten F. Does a 'Singing Together Group' improve the quality of life of people with a dementia and their carers? A pilot evaluation study. *Dementia* 2013;**12**:157–76. doi:10.1177/1471301211422761

20 Davidson JW, Almeida RA. An exploratory study of the impact of group singing activities on lucidity, energy, focus, mood and relaxation for persons with dementia and their caregivers. *Psychology of Well-Being* 2014;**4**:24. doi:10.1186/s13612-014-0024-5

For peer review only - http://bmjopen.bmj.com/site/about/guidelines.xhtml

21 Osman SE, Tischler V, Schneider J. 'Singing for the Brain': A qualitative study exploring the health and well-being benefits of singing for people with dementia and their carers. *Dementia* 2016;**15**:1326–39. doi:10.1177/1471301214556291

22 Hara M. Expanding a Care Network for People with Dementia and their Carers Through Musicking: Participant Observation with "Singing for the Brain." *1* 2011;**11**. doi:10.15845/voices.v11i2.570

23 Reagon C, Gale N, Dow R, *et al.* Choir singing and health status in people affected by cancer. *Eur J Cancer Care (Engl)* 2017;**26**. doi:10.1111/ecc.12568

24 Morris JR, Dow R. A model for the continuous quality improvement of the Sing With Us choir leaders. *Journal of Arts & Communities* 2014;**6**:133–144.

25 Zigmond AS, Snaith RP. The hospital anxiety and depression scale. *Acta Psychiatr Scand* 1983;**67**:361–70.

26 Tennant R, Hiller L, Fishwick R, *et al.* The Warwick-Edinburgh Mental Well-being Scale (WEMWBS): development and UK validation. *Health and Quality of Life Outcomes* 2007;**5**:63. doi:10.1186/1477-7525-5-63

27 Fancourt D, Perkins R, Ascenso S, *et al.* Effects of Group Drumming Interventions on Anxiety, Depression, Social Resilience and Inflammatory Immune Response among Mental Health Service Users. *PLOS ONE* 2016;**11**:e0151136. doi:10.1371/journal.pone.0151136

28 Warran K, Fancourt D, Wiseman T. How does the process of group singing impact upon people affected by cancer? A grounded theory study. *BMJ Open*

Figure 1: CONSORT diagram of participants

Figure 2: Predictive margins of time by group interaction with 95% confidence intervals for (A) symptoms of anxiety, (B) symptoms of depression, (C) wellbeing. Control group is shown as a hard line, and experimental group as a dashed line. The model is adjusted for demographic factors, health-related factors, cultural engagement and attitudes to singing, and length of time caring for somebody with cancer.

For peer review only

For peer review only - http://bmjopen.bmj.com/site/about/guidelines.xhtml

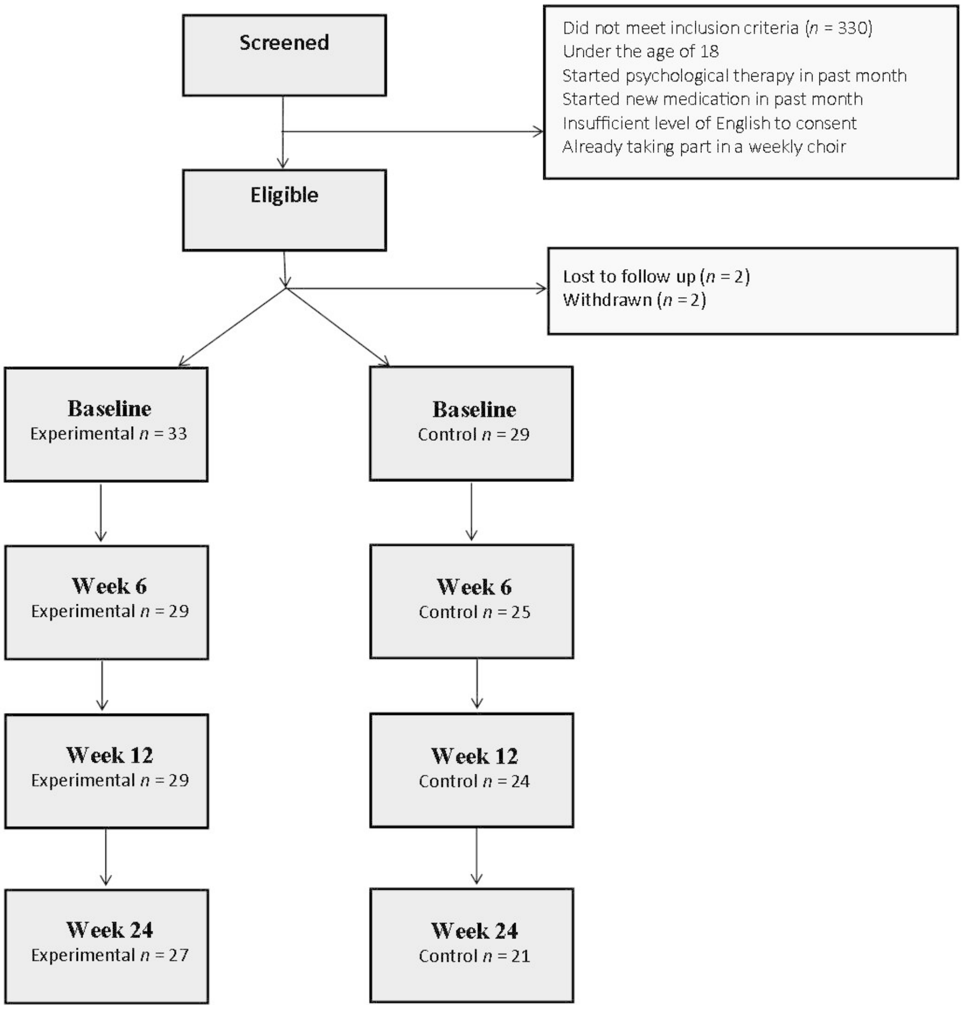

Figure 1: CONSORT diagram of participants

181x181mm (150 x 150 DPI)

Figure 2: Predictive margins of time by group interaction with 95% confidence intervals for (A) symptoms of anxiety, (B) symptoms of depression, (C) wellbeing. Control group is shown as a hard line, and experimental group as a dashed line.

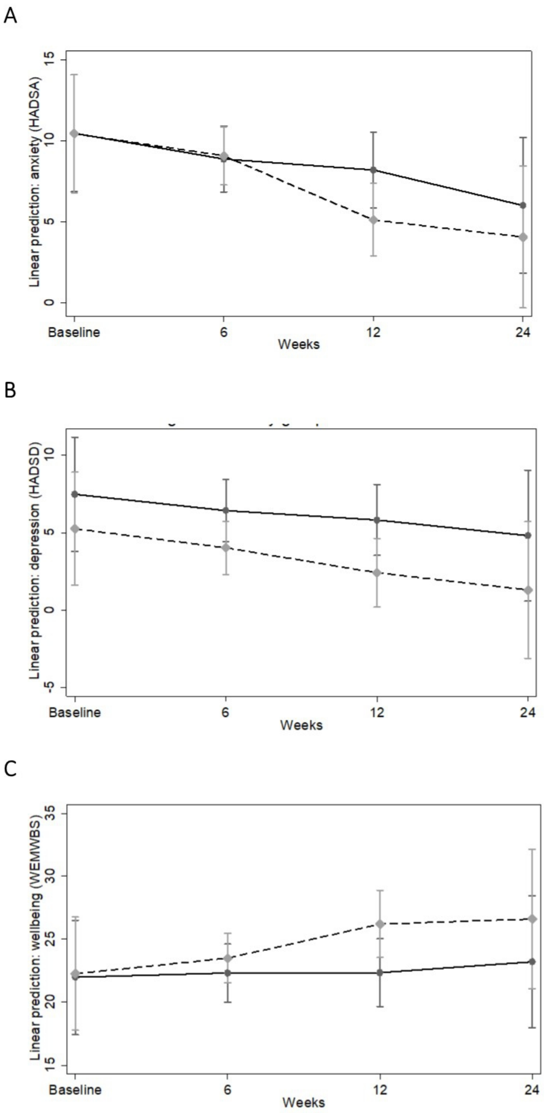

Figure 2: Predictive margins of time by group interaction with 95% confidence intervals for (A) symptoms of anxiety, (B) symptoms of depression, (C) wellbeing. Control group is shown as a hard line, and experimental group as a dashed line. The model is adjusted for demographic factors, health-related factors, cultural engagement and attitudes to singing, and length of time caring for somebody with cancer.

215x271mm (150 x 150 DPI)

BMJ Open

Psychosocial singing interventions for the mental health and wellbeing of family carers of cancer patients: results from a longitudinal controlled study

Journal:	*BMJ Open*
Manuscript ID	bmjopen-2018-026995.R2
Article Type:	Research
Date Submitted by the Author:	17-May-2019
Complete List of Authors:	Fancourt, Daisy; University College London Research Department of Epidemiology and Public Health, Department of Behavioural Science and Health<br>Warran, Katey; The University of Edinburgh, Edinburgh College of Art<br>Finn, Saoirse; Royal College of Music, Centre for Performance Science<br>Wiseman, Theresa; The Royal Marsden NHS Foundation Trust, ; University of Southampton, Faculty of Health Sciences
**Primary Subject Heading**:	Health services research
Secondary Subject Heading:	Complementary medicine, Mental health, Patient-centred medicine
Keywords:	Anxiety disorders < PSYCHIATRY, MENTAL HEALTH, Depression & mood disorders < PSYCHIATRY, SOCIAL MEDICINE, ONCOLOGY

SCHOLARONE™
Manuscripts

Psychosocial singing interventions for the mental health and wellbeing of family carers of cancer patients: results from a longitudinal controlled study

SHORT RUNNING TITLE: Psychosocial singing interventions for the mental health of cancer carers

Fancourt D[1]*, Warran K[2], Finn S[2], Wiseman T[3,4]

[1] Department of Behavioural Science and Health, University College London, UK

[2] Centre for Performance Science, Royal College of Music, London, UK

[3] The Royal Marsden NHS Foundation Trust, London, UK

[4] Faculty of Health Sciences, University of Southampton, Southampton, UK

* Corresponding author: Dr Daisy Fancourt, University College London, 1-19 Torrington Place, London WC1E 7HB, d.fancourt@ucl.ac.uk

Word count: 2099

For peer review only - http://bmjopen.bmj.com/site/about/guidelines.xhtml

ABSTRACT

Objective. The mental health challenges facing people who care for somebody with cancer are well documented. While many support interventions focus on provision of information or cognitive behavioural therapy, the literature suggests that psychosocial interventions could also be of value, especially given the low social support frequently reported by carers. Singing is a psychosocial activity shown to improve social support, increase positive emotions and reduce fatigue and stress. This study explored the effects of weekly group singing on anxiety, depression and wellbeing in cancer carers over a six-month period.

Design. A multi-site non-randomised longitudinal controlled study

Setting. The Royal Marsden NHS Trust in Greater London.

Participants. 62 adults who currently care for a spouse, relative or close friend with cancer who had not recently started any medication or psychological therapy.

Interventions. On enrolment, participants selected to join a weekly community choir for 12 weeks (n=33) or continue with life as usual (n=29).

Outcome measures. The primary outcome was mental health using the Hospital Anxiety and Depression Scale. Secondary outcomes were wellbeing using the Warwick Edinburgh Mental Wellbeing Scale. Using linear mixed effects models, we compared the change in mental health and wellbeing over time between the two groups while adjusting for confounding variables including demographics, health-related variables, musical engagement and length of time caring.

Results. Participants in the choir group showed a significantly greater decrease in anxiety over time than participants in the control group (B=-0.94, SE=0.38, p=.013), and a significantly greater increase in wellbeing (B=1.25, SE=0.49, p=.011). No changes were found for depression. Sub-group analyses showed carers with anxiety or below-average wellbeing were most likely to benefit.

Conclusions. This study builds on previous research showing the mental health benefits of singing for people with cancer by showing that weekly singing can also support anxiety and wellbeing in cancer carers.

KEYWORDS

Cancer, oncology, carers, anxiety, depression, wellbeing, singing, psychosocial, social support

For peer review only - http://bmjopen.bmj.com/site/about/guidelines.xhtml

ARTICLE SUMMARY

Strengths and limitations of this study

- We used random-intercept random-slope linear mixed effects models which allowed us to compare different rates of change in mental health and wellbeing over time amongst carers who did and didn't take part in a weekly choir over 24 weeks.
- Our results were found independent of identified confounding factors including demographics, health-related variables, musical engagement and length of time caring.
- This study had many strengths including its longitudinal tracking involving multiple time-points and its controlled design.
- Our groups were statistically well matched at baseline and results were not attenuated by the consideration of baseline confounders.
- However, our results were found in a relatively modest sample size, so future studies are required to ascertain whether results can be replicated in larger samples and our study was not randomised so we cannot assume full exchangeability between groups.

For peer review only

For peer review only - http://bmjopen.bmj.com/site/about/guidelines.xhtml

INTRODUCTION

Family members and close friends, particularly spouses, are often primary caregivers for individuals affected by cancer, playing a vital role in their care process. Yet, well documented across the literature are the psychological, physical, social and economic challenges faced by informal cancer carers [1,2]. In relation to *mental health*, carers have repeatedly been found to have greater anxiety and depression levels than general population controls [3,4]. Indeed, psychological distress in carers correlates with the distress experienced by patients and has been found through meta-analysis to be no different in size [5,6]. Prevalence rates of depression are around 26% in both patients and spouses and the prevalence of anxiety is around 28% in patients and 40% in spouses [7]. In particular, anxiety has been found to increase when caring for somebody with advanced cancer, exceeding depression in carers [8]. This has led to suggestions that anxiety, rather than depression, is the largest psychological problem in carers and calls for interventions that can tackle it [7].

In addition to mental health challenges, caring for somebody with cancer has also been found to affect *wellbeing*. A Europe-wide survey has found mental as well as physical health-related quality of life to be significantly lower in carers than non-caregivers [9]. Female carers in particular report lower quality of life than women who are not carers [5], and quality of life for carers of both genders has been found to vary across the illness trajectory [10]. Given that wellbeing is associated with mental health and also related factors such as resilience and coping, there is a need too for interventions that can support wellbeing in carers.

Many interventions for carers focus on the provision of information designed to directly support carers in their roles, or involve cognitive behavioural therapy to support and enhance coping [11,12]. A meta-analysis of therapeutic counselling, skills training and psychoeducational interventions for caregivers of cancer patients found evidence of reductions in caregiving burden, enhanced coping strategies, self-efficacy, physical functioning, anxiety, family relations and social functioning [13]. Interventions were identified as being more effective if caregivers attended alone (i.e. without the person they cared for) and if the interventions were delivered in a group setting with other carers. However, there has also been increasing interest in the role of psychosocial interventions for supporting mental health and wellbeing in carers (e.g. [14]. Such interest has been encouraged by research suggesting that, in addition to specific needs relating to their caregiving, carers frequently have unmet personal needs and low social support themselves [15]. In a population-based study of family carers of cancer survivors, social support was one of the

For peer review only - http://bmjopen.bmj.com/site/about/guidelines.xhtml

strongest predictors of quality of life [16]. Carers with less social support also have a higher risk of developing depression and anxiety [1]. Yet, caregivers often feel that they receive less support in comparison to how much patients feel that they receive [16].

Singing is a promising multimodal psychosocial intervention that has been used to support mental health and wellbeing in diverse populations, including amongst cancer patients [17]. In cancer caregivers, singing has been shown to increase perceived social support, enhance short-term positive emotions, reduce fatigue, promote relaxation and also reduce levels of stress hormones and mood-related inflammation over a single one hour singing session [18]. However, to date there is little research into whether singing can specifically reduce anxiety and depression and enhance wellbeing in cancer caregivers, and the effects of singing over longer periods of time. Whilst a few studies have identified benefits of singing for other groups of carers, such as carers of people with dementia, these studies have typically been very small in sample size (e.g. 4-7 participants completing data collection), followed participants over relatively short time frames (e.g. just 6 weeks), and employed little quantitative data [19–22]. Consequently, this study built on the theoretical rationale for the benefits of psychosocial interventions for cancer carers and the preliminary data showing the benefits of singing specifically for carer populations and explored whether weekly singing is linked with improvements in anxiety, depression and wellbeing in carers of somebody affected by cancer over a six-month period. We hypothesised that singing in a choir would be associated with improvements in all three outcomes, and that this response would be strongest amongst those with poorest mental health and wellbeing at baseline.

MATERIALS AND METHODS

Participants and procedure

This study was a multi-site non-randomised longitudinal controlled study. To be eligible, participants had to self-identify as currently caring for a spouse, relative or close friend with cancer. We excluded members of hospital staff in order to differentiate between formal and informal care. Participants were excluded if they were under the age of 18, if they were already engaged in a weekly group choir, if they had started a formal course of psychological therapy in the last month or were scheduled to start one in the next 12 weeks, if they had started any new medication for anxiety or depression in the last month, or if their level of English was insufficient to complete the questionnaires required. Participants were recruited through NHS hospital trusts in Greater London, carer support groups, community and

For peer review only

charity events and social media. A total of 396 were screened for enrolment, 66 were eligible and consented to take part, and 62 participants (29 control and 33 experimental) enrolled in the study (see Figure 1 for CONSORT diagram of participants). The study received ethical approval from the National Research Ethics Service [16/LO/0579] and all participants provided informed consent.

FIGURE 1 ABOUT HERE

Figure 1: CONSORT diagram of participants

On enrolment, participants were given the option of joining a weekly choir for 12 weeks. This length of study was selected from pilot data collected on singing for patients affected by cancer who were involved in uncontrolled studies in Wales, which suggested that improvements in mental health were found 12 weeks following joining, with further improvements by 24 weeks [23]. Participants who did not select to join a choir formed the control group and continued with life as usual. Participants who did select to join a choir took part in a 90-minute session each week led by a professional choir leader, of which 60 minutes was dedicated to singing (including warm-up exercises, learning new songs and singing familiar songs) and 30 minutes to socialising. The repertoire focused on popular songs arranged specifically for the choirs with backing tracks. All songs were learnt by ear without participants need to read music. These choirs were led by Tenovus Cancer Care, specifically for people affected in some way by cancer, and the format of the sessions has been described in previous studies [24]. Both choirs used in this study were established specifically for the purposes of collecting data. Following 12 weeks in the choir, participants were given the option to continue with weekly choir sessions if they wished, with 19 participants (58%) choosing to leave and the remaining 42% choosing to continue attending. Those who continued attending attended an average of 10 sessions out of 12, with 57% of them attending all 12. All participants were asked to complete questionnaires at baseline, 6 and 12 weeks as well as at 3-month follow-up (24 weeks).

Patient and Public Involvement

The research questions and outcome measures were selected with the support of a Patient and Public Involvement (PPI) group who were participants in the Tenovus Cancer Choirs in Wales. These participants also approved the design of the study. As this was the first study of the choirs at the Royal Marsden Hospital sites (or indeed in England) and there was therefore not data about the response from individuals in the community or the hospitals to the choirs, it

For peer review only - http://bmjopen.bmj.com/site/about/guidelines.xhtml

was proposed by our PPI group that the study could be run as a longitudinal controlled study rather than a randomised controlled trial (RCT). As such, any carers would have the option of joining immediately rather than becoming part of a wait-list control. Whilst this has obvious methodological limitations, it was felt to be an important precursor to an RCT that would enable an assessment of the reception of the choirs in these new locations alongside measurements of outcome data. Participants in the study also supported recruitment efforts by helping to identify further participants. Dissemination plans include presentations back to members of all of the Tenovus Cancer Choirs in Wales and England.

Measures

Our primary outcomes were anxiety and depression, measured using the Hospital Anxiety and Depression Scale (HADS), which contains two independent subscales: one for anxiety and one for depression ranging from 0-21 for each construct with higher scores indicating poorer mental health (Bjelland, Dahl, Haug, & Neckelmann, 2002). When using this measure, scores of 0-7 are considered as non-indicative of anxiety or depression, while scores of 8-10 present mild cases, 11-14 present moderate cases, and 15-21 present severe cases [25].

Our secondary outcome was wellbeing, measured using the Warwick Edinburgh Mental Wellbeing Scale (WEMWBS) short form, which encompasses both hedonic and eudemonic wellbeing [26]. The scale is scored from 7-35 with higher scores representing higher levels of wellbeing. As recommended in validations, the raw scores were logit transformed prior to analysis [27]. The New Economics Foundation suggests five levels of wellbeing based on quintile analyses of data in the UK Understanding Society Survey, 2009: poor (<22), below average (22–24), average (25–26), good (27–28) and excellent (29– 35).

Additionally, we collected data on age, gender, income (<£16,000, £16,000-£30,000, £31,000-£60,000, £61,000-£90,000, >£90,000), employment status (unemployed, voluntary/temporary work, part-time work, full-time work, retired), whether participants were receiving psychological therapy, whether participants had any other health condition, whether participants had previously sung in a choir, how confident participants felt about singing (from 1-not at all to 5-very), whether participants had attended musical concerts or performances in the past year, whether they had taken part in any other music activity in the past year, and how long participants had been in a caring role for.

Statistics

For peer review only - http://bmjopen.bmj.com/site/about/guidelines.xhtml

To compare the choir vs the control group at baseline, we used one-way analyses of variance, and Fisher's exact tests. To explore changes in mental health and wellbeing over time and between groups, we used linear mixed effects models (LMMs). LMMs allow for natural heterogeneity amongst participants and, unlike some other repeated measures analysis models such as repeated measures analyses of variance which deal with missing data through list-wise deletion, linear mixed effects models make full use of the dataset. Our participants provided data an average of 3.4 times across the four time points, providing 180 data points. Our models used a random intercept and random slope, with an unstructured covariance matrix of the random effects. Time was modelled first as both a continuous variable to identify an overall time*group relationship (using a linear model as we found no evidence to support the use of a quadratic model), and then time was modelled as four separate time points to identify where specific changes occurred. We tested quadratic models but found no evidence to support their usage over a linear model. All models demonstrated normality of residuals and no evidence of heteroscedasticity.

We built up our final model through considering nested models of relevant covariates. Model 1 was unadjusted apart from baseline mental health, model 2 adjusted for demographic variables (age, gender, income and employment status), model 3 additionally adjusted for health-related variables (undergoing psychological therapy, and any other health conditions), model 4 additionally adjusted for cultural engagement and attitudes to singing (past experiences singing in a choir, confidence in singing, attendance at concerts or performances in the past year, and engagement in any other musical activity in the past year), and model 5 additionally adjusted for length of time caring for somebody with cancer. We used the log-likelihood ratio, Akaike's information criterion and Bayesian information criterion to confirm best fit of model 5. We calculated margins of response from the fully-adjusted model and created profile plots to illustrate the time*group interactions. As three outcomes were considered, a Bonferroni's alpha of 0.05/3=0.017 can be assumed. Standardised residuals were generated to confirm the assumption of normality.

In order to ascertain whether those with poorer mental health and wellbeing were more or less likely to benefit from engagement, sub-group analyses excluded those with no evident mental health problems at baseline. Additionally, given participants had been given the option of whether to continue with weekly singing following the first 12 weeks, we statistically compared differences in demographics and mental health amongst those who did and did not continue and also ran sensitivity analyses additionally adjusting for singing patterns after the initial 12 weeks to see if these affected outcomes at 24 weeks. All results shown are fully-adjusted models (i.e. Model 5) and all analyses were conducted using Stata v14.

For peer review only - http://bmjopen.bmj.com/site/about/guidelines.xhtml

197
198
199 **RESULTS**

200 **Demographics**

201 Participants were well matched at baseline on all demographic variables including mental health and wellbeing, with
202 no difference in baseline levels of anxiety or depression or wellbeing. The only significant difference between groups
203 was in confidence in singing, with the control group showing lower overall confidence than the singing group (see
204 Table 1).

205 Table 1: Baseline demographics of participants

	Control (n=29)	Choir (n=33)	P value
Age, mean (SD)	51 (15)	58 (14)	0.075 [a]
Sex, % female	72.4%	81.8%	0.54 [b]
Income, %			0.076 [b]
<£16,000	12.0%	20.7%	
£16,000-£30,000	24.0%	41.4%	
£31,000-£60,000	32.0%	24.1%	
£61,000-£90,000	32.0%	6.9%	
>£91,000	0%	6.9%	
Employment status, %			0.22 [b]
Unemployed	13.8%	3.2%	
Voluntary	0%	6.5%	
Part-time work	20.7%	29.0%	
Full-time work	37.9%	22.6%	
Retired	27.6%	38.7%	
Currently having therapy, %	10.3%	24.2%	0.19 [b]
Health condition, %	72.4%	60.6%	0.42 [b]
Previously sung in a choir, %	65.5%	48.5%	0.21 [b]
Confidence in singing, %			**0.040** [b]
1 – not at all	50%	21.2%	
2	21.4%	12.1%	
3	21.4%	36.4%	
4	3.6%	21.2%	
5 – very	3.6%	9.1%	
Attended a concert or performance in the past month, %	32.1%	51.5%	0.19 [b]
Took part in a music activity in the past month, %	18.5%	36.4%	0.16 [b]
Length of time being a carer, %			0.45 [b]
0-3 months	0%	9.7%	
3-12 months	17.9%	19.4%	
1-3 years	50%	35.5%	
3-5 years	14.3%	22.6%	
5+ years	17.9%	12.9%	
Anxiety, mean (SD)	8.04 (4.71)	8.97 (4.19)	0.42 [a]
Depression, mean (SD)	5.43 (4.49)	4.88 (3.08)	0.57 [a]
Wellbeing, mean (SD)	23.15 (4.36)	21.71 (3.70)	0.17 [a]

206 [a] one-way ANOVA; [b] Fisher's exact test

For peer review only - http://bmjopen.bmj.com/site/about/guidelines.xhtml

For peer review only

Anxiety

At baseline, 44.3% (n=27) of participants showed no anxiety (HADS Anxiety (HADSA) score 0-7), 21.3% (n=13) showed mild anxiety (HADSA score 8-10), 23.0% (n=14) showed moderate anxiety (HADSA score 11-14), and 11.5% (n=7) showed severe anxiety (HADSA score 15-21). The linear mixed effects model with time as a continuous variable showed that there was a significant time by group interaction, with participants in the choir group showing a significantly greater decrease in anxiety than participants in the control group (B=-0.94, SE=0.38, p=.013). When modelling time as four separate time points, this difference was not apparent in the first 6 weeks (B=0.22, SE=0.81, p=.78), but was apparent by week 12 (B=-3.06, SE=0.92, p=.001), with the difference between groups decreasing slightly by week 24 (B=-1.93, SE=1.12, p=.086) (see Figure 2A). In the choir group, this equated to 17.5% decrease in anxiety symptoms across the first 12 weeks, which had slightly decreased to an 11.9% decrease in anxiety symptoms by 24 weeks.

Depression

At baseline, just 14.8% (n=9) of participants showed mild depression and only 11.5% (n=7) showed moderate depression, with no participants showing severe depression. There was no evidence of a time by group interaction for depression with time as a continuous variable (B=-0.49, SE=0.39, p=.21). When modelling time as four separate time points, there was similarly no difference evident at 6 weeks (B=-0.18, SE=0.81, p=.82), 12 weeks (B=-1.17, SE=0.94, p=.22), or 24 weeks (B=-1.28, SE=1.19, p=.28) (see Figure 2B).

Wellbeing

At baseline, 52.5% (n=32) of participants had poor wellbeing (<22), 16.4% (n=10) were below average (22–24), 16.4% (n=10) were average (25–26), 8.2% (n=5) were good (27–28) and 6.6% (n=4) were excellent (29– 35). There was a significant time by group interaction for wellbeing when modelling time as a continuous variable, with participants in the choir group showing a significantly greater increase in wellbeing than participants in the control group (B=1.25, SE=0.49, p=.011). When modelling time as four separate time points, this difference was not apparent in the first 6 weeks (B=0.89, SE=0.98, p=.36), but was apparent by week 12 (B=3.58, SE=1.17, p=.002), and was still present by week 24 (B=3.10, SE=1.50, p=.038) (see Figure 2C). This equated to a 12.6% increase in wellbeing in the choir group across the first 12 weeks, which had slightly decreased to a 10.5% increase by week 24.

FIGURE 2 ABOUT HERE

For peer review only - http://bmjopen.bmj.com/site/about/guidelines.xhtml

Figure 2: Predictive margins of time by group interaction with 95% confidence intervals for (A) symptoms of anxiety, (B) symptoms of depression, (C) wellbeing. Control group is shown as a hard line, and experimental group as a dashed line. The model is adjusted for demographic factors, health-related factors, cultural engagement and attitudes to singing, and length of time caring for somebody with cancer.

Sub-group and sensitivity analyses

We used sub-group analyses to test whether participants in the choir group demonstrating anxiety and/or low wellbeing at baseline showed improvements over the 24 weeks compared to those in the control group.

Anxiety

As a first sub-group analysis, we excluded participants with no evidence of anxiety at baseline (HADSA score<8) and re-ran analyses (n=27; control group=16, choir group=18). This confirmed a significant time by group interaction for anxiety, with participants in the choir group showing a significantly greater decrease in anxiety than participants in the control group (B=-2.62, SE=0.58, p<.001). This difference was not apparent in the first 6 weeks, was was apparent by week 12 (B=-7.47, SE=1.28, p<.001), and was maintained at week 24 (B=-5.57, SE=1.64, p=.001).

These participants with anxiety also showed evidence of improvements in wellbeing, with a significant time by group interaction (B=1.93, SE=0.57, p=.001). As with full-sample wellbeing analyses, this difference was not apparent in the first 6 weeks (B=0.25, SE=1.08, p=0.81), but was apparent by week 12 (B=5.12, SE=1.27, p<.001), and was maintained at week 24 (B=4.32, SE=1.68, p=.01).

Wellbeing

As a second sub-group analysis, we excluded participants with average or above average wellbeing at baseline (WEMWBS score>25) and re-ran analyses (n=42; control group=18, choir group=24). This confirmed a significant time by group interaction for wellbeing, with participants in the choir group showing a significantly greater improvement in wellbeing than participants in the control group (B=1.30, SE=0.51, p=.01). This difference was not apparent in the first 6 weeks (B=1.45, SE=1.00, p=.15), but was apparent by week 12 (B=1.45, SE=1.16, p<.001), although it was slightly attenuated at week 24 (B=2.59, SE=1.52, p=.087).

These participants with low wellbeing also showed evidence of improvements in anxiety, with a significant time by group interaction (B=-1.22, SE=0.51, p=.017). As with full-sample anxiety analyses, this difference was not apparent

For peer review only - http://bmjopen.bmj.com/site/about/guidelines.xhtml

For peer review only

in the first 6 weeks (B=0.95, SE=0.96, p=0.33), but was apparent by week 12 (B=-3.91, SE=1.10, p<.001), although it was slightly attenuated by week 24 (B=-2.34, SE=1.41, p=.097).

Singing continuation

We compared differences between those who did and did not continue with singing beyond the first 12 weeks. There were no demographic differences between groups or differences in mental health between those who continued with the singing following week 12. As a sensitivity analysis, we additionally factored in whether participants continued to sing beyond the first 12 weeks on our primary and secondary outcomes. Singing behaviours after the initial 12 weeks were found to not affect the significance of overall results nor significantly predict trajectories in anxiety, depression or wellbeing.

For peer review only

DISCUSSION

This study explored whether singing in a choir is linked with improvements in mental health and wellbeing in people who care for somebody with cancer. Results partly confirmed our hypotheses, showing that singing in a choir on a weekly basis was significantly associated with improvements in anxiety and wellbeing, but not depression. These results were independent of demographic covariates, health-related covariates, previous or current engagement in musical activities, attitudes to singing and length of time caring. As predicted, these improvements in mental health and wellbeing were found amongst carers generally, as well as specifically carers with anxiety or below-average wellbeing. Differences between groups were not found within the first 6 weeks but were found by week 12 and were generally maintained at the 3-month follow-up. 42% of carers involved in the choir group continued to sing in the follow-up period. However, continuing to sing beyond the initial 12 week period did not significantly predict wellbeing or anxiety at follow-up.

Our findings are in line with meta-analyses suggesting that anxiety rather than depression is the major challenge amongst cancer carers [7]. In total, 56% of our participants showed symptoms of anxiety, with 35% specifically showing symptoms of moderate or severe anxiety. The finding that singing was associated with a greater decrease in anxiety echoes findings from previous studies of singing amongst people affected by cancer. In a study of 143 non-patients affected by cancer (comprising carers, staff and those who had been bereaved), singing reduced levels of anxiety but not depression using HADS over a 6-month period [23]. Further, our finding suggesting benefits continued

For peer review only - http://bmjopen.bmj.com/site/about/guidelines.xhtml

to be felt regardless of whether participants sung after the initial 12 weeks mirrors results from group drumming interventions for people with mild-moderate mental health conditions, which similarly found a maintenance of mental health benefits 3 months following the end of a 10-week programme [28].

A key consideration is how choir singing might reduce anxiety. A separate grounded theory study of the choirs involved in this study identified four key mechanisms [29]. First, the choirs provided emotional and uplifting experiences that participants experienced as a mind-body activity that supported their sense of identity. Second, the choirs provided social support; specifically an 'unspoken' group support that enabled the development of friendships, the perception of a caring network, and a sense of inspiration from the choir leader. Third, the choirs helped to build resilience amongst participants, developing coping skills, building confidence and leading to wider social and behavioural changes in individual's lives beyond the individual rehearsals themselves. Finally, the choirs provided members with the chance to develop musical skills which enhanced their sense of self-esteem. In particular, the social support provided by the choirs may have been a key component for the carers in this study, as social support has been found to be a significant predictor of anxiety and quality of life in caregivers [1,16].

Strengths and limitations

This study had many strengths including its longitudinal tracking involving multiple time-points and its controlled design. We also considered factors such as additional psychological support being received by participants and deliberately only included participants who had not recently had any change in their psychological support or medication. The study was not randomised, so while this did have the advantage of mimicking real life choice in that participants had the option of selecting whether to enrol in the choir or not, it means that causality cannot be assumed. Notably, we used a statistical approach that allowed us to model the effects of variables that could have affected exchangeability between groups on mental health trajectories across the 24 weeks,our groups were extremely well matched statistically at baseline, and results were not attenuated by the consideration of baseline confounders. But future randomised controlled trials are required to confirm whether involvement in choirs is causally linked with mental health. Further, there was a significant difference at baseline in terms of confidence in singing, with those who joined the choir professing to be more confident. Whilst we factored this difference into our analyses such that it cannot explain the results, it does suggest that singing interventions may not be suitable for all carers or may need adaptations in order to attract and retain those who feel less confident. Our results were also found in a relatively modest sample size, so future studies are required to ascertain whether results can be replicated in larger samples.

For peer review only - http://bmjopen.bmj.com/site/about/guidelines.xhtml

Future studies could benefit from focusing more specifically on particular points in caregiving (such as around initial diagnosis or during palliative care). Such studies could also focus specifically on carers with known mental health diagnoses such as moderate anxiety, especially given the findings here that singing was of benefit both to those with and without baseline anxiety or low wellbeing. Finally, this study focused exclusively on carers of somebody with cancer. However, whether the longitudinal benefits of group singing suggested here are replicated amongst other carer groups remains to be explored.

Conclusions

In conclusion, this study builds on previous research showing the mental health benefits of singing for people affected by cancer by showing that weekly singing may also support anxiety and wellbeing in carers. Given that singing is a simple community activity free from the stigma that sometimes surrounds engagement with more formal support services, it could be promoted to support the mental health and wellbeing of cancer caregivers. These results suggests the value of conducting future randomised controlled studies.

COMPETING INTERESTS

The funding for this study was provided by Tenovus Cancer Care who run the choirs used as the intervention in this study. However, no member of staff from Tenovus Cancer Care was involved in the specifics of the study design or in the data collection, analysis or interpretation of the data, the writing of the report, or the decision to submit the paper for publication.

FUNDING

This research was funded by Tenovus Cancer Care [SWU2016-01]. DF is funded by the Wellcome Trust [205407/Z/16/Z]. TW is funded by the Royal Marsden Cancer Charity. The research was facilitated by the infrastructure of the RM/ICR NIHR Biomedical Research Centre. The study team acknowledge the support of the National Institute of Health Research Clinical Research Network (NIHR CRN).

AUTHOR CONTRIBUTORSHIP STATEMENT

For peer review only - http://bmjopen.bmj.com/site/about/guidelines.xhtml

DF and TW designed the study. SF and KW collected the data. DF analysed the data and drafted the manuscript. All authors critically appraised the manuscript and approved it for publication.

DATA SHARING STATEMENT

De-identified participant data are available for viewing or verification from the authors on request through contacting the corresponding author. Further analysis of the data for new research is not permitted by external parties under our ethical approval.

REFERENCES

1 Girgis A, Lambert S, Johnson C, *et al.* Physical, psychosocial, relationship, and economic burden of caring for people with cancer: a review. *J Oncol Pract* 2013;**9**:197–202. doi:10.1200/JOP.2012.000690

2 Northouse LL, Katapodi MC, Schafenacker AM, *et al.* The impact of caregiving on the psychological well-being of family caregivers and cancer patients. *Semin Oncol Nurs* 2012;**28**:236–45. doi:10.1016/j.soncn.2012.09.006

3 Stenberg U, Ruland CM, Miaskowski C. Review of the literature on the effects of caring for a patient with cancer. *Psychooncology* 2010;**19**:1013–25. doi:10.1002/pon.1670

4 Trevino KM, Prigerson HG, Maciejewski PK. Advanced cancer caregiving as a risk for major depressive episodes and generalized anxiety disorder. *Psychooncology* 2018;**27**:243–9. doi:10.1002/pon.4441

5 Hagedoorn M, Buunk BP, Kuijer RG, *et al.* Couples dealing with cancer: role and gender differences regarding psychological distress and quality of life. *Psychooncology* 2000;**9**:232–42.

6 Hodges LJ, Humphris GM, Macfarlane G. A meta-analytic investigation of the relationship between the psychological distress of cancer patients and their carers. *Soc Sci Med* 2005;**60**:1–12. doi:10.1016/j.socscimed.2004.04.018

7 Mitchell AJ, Ferguson DW, Gill J, *et al.* Depression and anxiety in long-term cancer survivors compared with spouses and healthy controls: a systematic review and meta-analysis. *The Lancet Oncology* 2013;**14**:721–32. doi:10.1016/S1470-2045(13)70244-4

8 Burridge LH, Barnett AG, Clavarino AM. The impact of perceived stage of cancer on carers' anxiety and depression during the patients' final year of life. *Psycho-Oncology*;**18**:615–23. doi:10.1002/pon.1435

9 Goren A, Gilloteau I, Lees M, *et al.* Quantifying the burden of informal caregiving for patients with cancer in Europe. *Support Care Cancer* 2014;**22**:1637–46. doi:10.1007/s00520-014-2122-6

10 Kim Y, Given BA. Quality of life of family caregivers of cancer survivors. *Cancer*;**112**:2556–68. doi:10.1002/cncr.23449

11 Frambes D, Given B, Lehto R, *et al.* Informal Caregivers of Cancer Patients: Review of Interventions, Care Activities, and Outcomes. *West J Nurs Res* 2018;**40**:1069–97. doi:10.1177/0193945917699364

For peer review only - http://bmjopen.bmj.com/site/about/guidelines.xhtml

For peer review only

12 Pitceathly C, Maguire P. The psychological impact of cancer on patients' partners and other key relatives: a review. *European Journal of Cancer* 2003;**39**:1517–24. doi:10.1016/S0959-8049(03)00309-5

13 Northouse LL, Katapodi MC, Song L, *et al.* Interventions with family caregivers of cancer patients: meta-analysis of randomized trials. *CA Cancer J Clin* 2010;**60**:317–39. doi:10.3322/caac.20081

14 Badr H, Krebs P. A systematic review and meta-analysis of psychosocial interventions for couples coping with cancer. *Psychooncology* 2013;**22**:1688–704. doi:10.1002/pon.3200

15 Aoun SM, Kristjanson LJ, Currow DC, *et al.* Caregiving for the terminally ill: at what cost? *Palliat Med* 2005;**19**:551–5. doi:10.1191/0269216305pm1053oa

16 Mellon S, Northouse LL, Weiss LK. A population-based study of the quality of life of cancer survivors and their family caregivers. *Cancer Nurs* 2006;**29**:120–31; quiz 132–3.

17 Fancourt D, Warran K. Singing for cancer: implications from psychoneuroimmunology. In: *Advances in Interdisciplinary Research in Singing*. Routledge 2018.

18 Fancourt D, Williamon A, Carvalho LA, *et al.* Singing modulates mood, stress, cortisol, cytokine and neuropeptide activity in cancer patients and carers. *ecancermedicalscience* 2016;**10**. doi:10.3332/ecancer.2016.631

19 Camic PM, Williams CM, Meeten F. Does a 'Singing Together Group' improve the quality of life of people with a dementia and their carers? A pilot evaluation study. *Dementia* 2013;**12**:157–76. doi:10.1177/1471301211422761

20 Davidson JW, Almeida RA. An exploratory study of the impact of group singing activities on lucidity, energy, focus, mood and relaxation for persons with dementia and their caregivers. *Psychology of Well-Being* 2014;**4**:24. doi:10.1186/s13612-014-0024-5

21 Osman SE, Tischler V, Schneider J. 'Singing for the Brain': A qualitative study exploring the health and well-being benefits of singing for people with dementia and their carers. *Dementia* 2016;**15**:1326–39. doi:10.1177/1471301214556291

22 Hara M. Expanding a Care Network for People with Dementia and their Carers Through Musicking: Participant Observation with "Singing for the Brain." *1* 2011;**11**. doi:10.15845/voices.v11i2.570

23 Reagon C, Gale N, Dow R, *et al.* Choir singing and health status in people affected by cancer. *Eur J Cancer Care (Engl)* 2017;**26**. doi:10.1111/ecc.12568

24 Morris JR, Dow R. A model for the continuous quality improvement of the Sing With Us choir leaders. *Journal of Arts & Communities* 2014;**6**:133–144.

25 Zigmond AS, Snaith RP. The hospital anxiety and depression scale. *Acta Psychiatr Scand* 1983;**67**:361–70.

26 Tennant R, Hiller L, Fishwick R, *et al.* The Warwick-Edinburgh Mental Well-being Scale (WEMWBS): development and UK validation. *Health and Quality of Life Outcomes* 2007;**5**:63. doi:10.1186/1477-7525-5-63

27 Stewart-Brown S, Tennant A, Tennant R, *et al.* Internal construct validity of the Warwick-Edinburgh Mental Well-being Scale (WEMWBS): a Rasch analysis using data from the Scottish Health Education Population Survey. *Health and Quality of Life Outcomes* 2009;**7**:15. doi:10.1186/1477-7525-7-15

28 Fancourt D, Perkins R, Ascenso S, *et al.* Effects of Group Drumming Interventions on Anxiety, Depression, Social Resilience and Inflammatory Immune Response among Mental Health Service Users. *PLOS ONE* 2016;**11**:e0151136. doi:10.1371/journal.pone.0151136

29 Warran K, Fancourt D, Wiseman T. How does the process of group singing impact upon people affected by cancer? A grounded theory study. *BMJ Open*

For peer review only

For peer review only - http://bmjopen.bmj.com/site/about/guidelines.xhtml

Figure 1: CONSORT diagram of participants

Figure 2: Predictive margins of time by group interaction with 95% confidence intervals for (A) symptoms of anxiety, (B) symptoms of depression, (C) wellbeing. Control group is shown as a hard line, and experimental group as a dashed line. The model is adjusted for demographic factors, health-related factors, cultural engagement and attitudes to singing, and length of time caring for somebody with cancer.

For peer review only

For peer review only - http://bmjopen.bmj.com/site/about/guidelines.xhtml

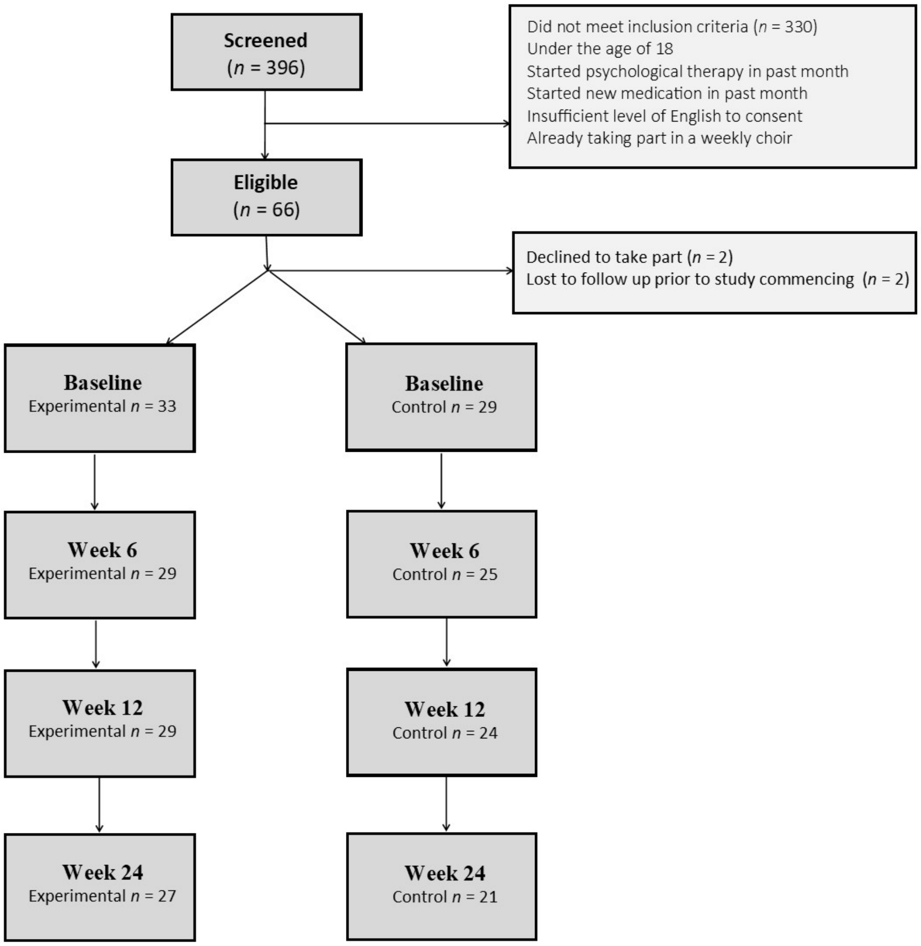

Figure 1

193x191mm (150 x 150 DPI)

Figure 2: Predictive margins of time by group interaction with 95% confidence intervals for (A) symptoms of anxiety, (B) symptoms of depression, (C) wellbeing. Control group is shown as a hard line, and experimental group as a dashed line.

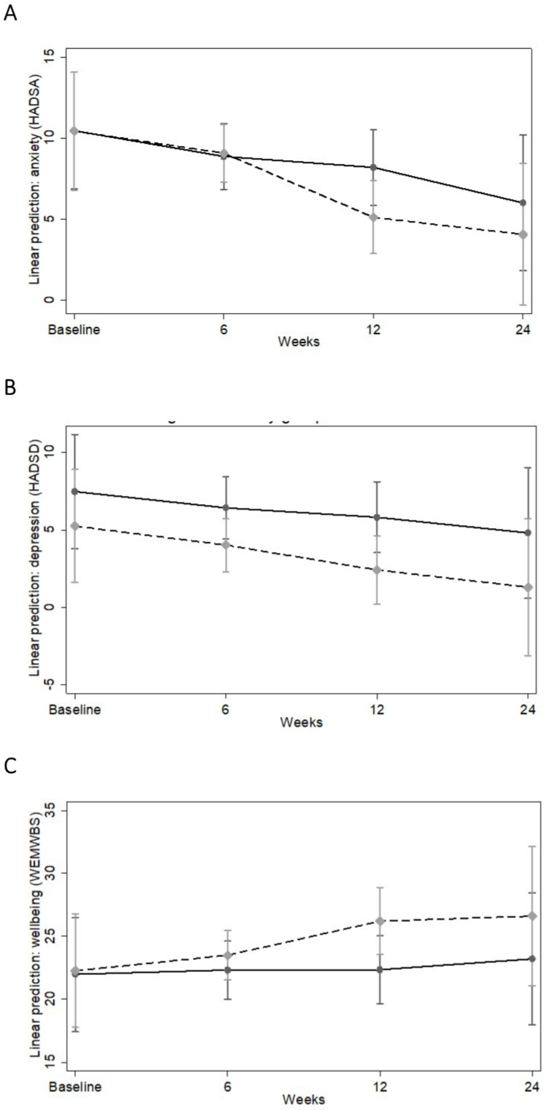

Figure 2: Predictive margins of time by group interaction with 95% confidence intervals for (A) symptoms of anxiety, (B) symptoms of depression, (C) wellbeing. Control group is shown as a hard line, and experimental group as a dashed line. The model is adjusted for demographic factors, health-related factors, cultural engagement and attitudes to singing, and length of time caring for somebody with cancer.

215x271mm (150 x 150 DPI)
